# Three-dimensional kinematics of the craniocervical junction of Cavalier King Charles Spaniels compared to Chihuahuas and Labrador retrievers

**DOI:** 10.1371/journal.pone.0278665

**Published:** 2023-01-17

**Authors:** Mareike-Kristin Nickel, Lisa Schikowski, Martin Stephan Fischer, Nicola Kelleners, Martin Jürgen Schmidt, Nele Eley

**Affiliations:** 1 Department of Veterinary Clinical Sciences, Small Animal Clinic–Surgery, Justus-Liebig-University, Giessen, Germany; 2 Institute of Zoology and Evolutionary Research, Friedrich-Schiller-University, Jena, Germany; 3 Department of Veterinary Clinical Sciences, Small Animal Clinic–Neurosurgery, Neuroradiology and Clinical Neurology, Justus-Liebig-University, Giessen, Germany; Graduate Career Consulting LLC, UNITED STATES

## Abstract

Our knowledge about the underlying pathomechanisms of craniocervical junction abnormalities (CCJA) in dogs mostly derives from measurements based on tomographic imaging. These images are static and the positioning of the dogs’ head does not reflect the physiological *in vivo* position of the craniocervical junction (CCJ). Aberrant motion patterns and ranges of motion (ROM) in sound individuals of CCJA predisposed breeds may be a pathogenetic trigger. To further extend our limited knowledge of physiological motion of the CCJ, this prospective, comparative study investigates the *in vivo* motion patterns and ROM of the CCJ in walk and trot in sound Cavalier King Charles Spaniels and Chihuahuas. The Labrador retriever is used as a reference breed without predisposition for CCJA. This is the first detailed description of CCJ movement of trotting dogs. Biplanar fluoroscopy images, recorded in walking and trotting dogs, were matched to a virtual reconstruction of the skull and cranial cervical spine utilising Scientific Rotoscoping. Kinematic data reveal the same motion patterns among all breeds and gaits with individual temporal and spatial differences in each dog. A stride cycle-dependent lateral rotation of the cranial cervical spine and axial rotation of the atlantoaxial joint in trot in dogs is described for the first time. The ROM of the atlantoaxial and atlantooccipital joints in walk and trot were not statistically significantly greater in the CCJA-predisposed breeds CKCS and Chihuahua. ROM values of all translational and rotational degrees of freedom were larger in walk than trot, although this is only statistically significant for the atlantoaxial joint. Until proven otherwise, a more species-specific than breed-specific general motion pattern of the CCJ in walking and trotting, clinically sound dogs must be assumed. Species-specific anatomic properties of the CCJ seem to supersede breed-specific anatomical differences in clinically sound dogs.

## Introduction

Craniocervical junction abnormalities (CCJA) in dogs derive from maldevelopment of the skull base that results in conformational anomalies of the occipital bone and vertebrae of the craniocervical junction (CCJ) [1–3]. A premature closure of the spheno-occipital synchondrosis was found to cause shortening of the cranial base in the Cavalier King Charles spaniel (CKCS) [4, 5]. As a consequence of the shortened skull base, the position of the basioccipital bone is further rostrally and the supraoccipital bone projects caudally over the dorsal arch of the atlas [6]. This also changes the conformation of the atlantooccipital joint and forces the atlas into a straighter position in relation to the occiput and axis [6]. The dorsal lamina of the atlas shifts caudally and ventrally against the spinous process of the axis. This conformation may be associated with thickening of the interarcuate ligament (atlantoaxial band), which has been interpreted as an indicator for instability in the atlantoaxial joint [7–9]. On the other hand, the rostral lamina of the atlas can intrude through the foramen magnum and into the caudal fossa. The overlap and repositioning of the atlas are often associated with subluxation of the dens of the axis producing compression and elevation of the spinal cord (basilar impression) [6, 7, 10, 11]. Shortening of the cranial base also results in a distortion of brain morphology and reduction of cranial capacity [12, 13], contributing to imaging findings related to Chiari-like malformation (CLM). The cerebellum is shifted under the occipital lobe and becomes compressed, causing crowding of the foramen magnum and cerebrospinal fluid (CSF) flow abnormalities [3]. Obliteration of the CSF cisterns and the subarachnoid space at the craniocervical transition causes turbulent flow and pressure waves of CSF within the subarachnoid space of the spinal cord, which results in syringomyelia (SM) and progressive spinal cord atrophy [14].

The relative position of the cranial cervical vertebra in dogs can vary considerably with positioning of the animals in dorsal or ventral recumbency [15]. Cerebellar compression caused by impingement of the atlas appears to be accentuated by positioning in hyperextension of the atlantooccipital joint [7, 15]. Knowledge about the normal position and breed-specific movement of the cranial cervical spine and CCJ in dogs is limited [16, 17], which further complicates a clear diagnosis and assessment of severity of instability of the CCJ [7]. In-depth knowledge about the physiological conformation and kinematic analysis of the head and CCJ in dogs in general, and in the CKCS in particular, is necessary to define breed-specific physiological ROM as well as potential individual variances.

Kinematic gait analysis deals with spatial and temporal parameters of motion like stride length or joint angles, independent of body mass and forces [18, 19]. Scientific Rotoscoping is a markerless and therefore non-invasive kinematic gait analysis method of XROMM methodology (X-Ray Reconstruction of Moving Morphology). The technique merges motion data from biplanar X-ray videos with skeletal morphology data from CT scans and creates 3D bone animations moving in 3D space. Scientific Rotoscoping is considered a methodology generating anatomically highly accurate animations, also for use in dogs and the spine in particular [20–22]. Highly precise and accurate motion measurements are enabled, although the technique is very elaborate and time consuming [20, 21]. A previous study of our research group was the first to describe the physiological in-vivo motion of the cranial cervical spine and CCJ in clinically sound, walking dogs [23]. In this previous study, dogs of a CCJA-predisposed breed, the Chihuahua, were compared to individuals of a non-predisposed breed, the Labrador retriever, and physiological motion patterns and ROM were presented. Despite size- and body conformation-related differences, the same basic motion patterns in walk were found in both breeds [23]. The objective of the current study was now to extend and reevaluate our knowledge about physiological motion patterns and ROM of the CCJ in dogs by investigating another CCJA-predisposed breed of interest, the CKCS. We first describe in detail the kinematics of the CCJ in trot in Chihuahuas, Labrador retrievers and CKCSs, as well as naturally occurring ROM of the CCJ in these breeds during locomotion. We hypothesize that sound individuals of CCJA predisposed breeds show larger ROM of the atlantoaxial and atlantooccipital joints. Positions of the atlas and axis in relation to each other and to the skull base in a physiological position during locomotion in all aforementioned breeds are compared to existing quantitative measurements that characterise CCJ instability. In addition, we evaluated the potential observer variability for the measured values to assess the validity of the methodology.

## Materials and methods

### Animals

Fifteen privately owned CKCSs and 15 privately owned Chihuahuas, representing the group of breeds predisposed to CCJA, and 14 privately owned Labrador retrievers, as a reference breed not predisposed to CCJA, were prospectively examined. Exclusion criteria were abnormal findings in the clinical, orthopaedic and neurological examination, pathological findings in cross-sectional imaging (see below), insufficient habituation to the treadmill, excessive arbitrary head movements during walking on the treadmill or incomplete data acquisition. Based on these criteria, 23 dogs were excluded from the study group.

Eight CKCSs, eight Chihuahuas and three Labrador retrievers were investigated in walk and eight CKCSs, four Chihuahuas and three Labrador retrievers in trot. We used the animation raw data of the Chihuahuas in Schikowski’s study [23]. The Labrador retrievers were the same dogs as in Schikowski’s study as well, but Scientific Rotoscoping and all calculations were obtained anew in order to test interobserver reliability. The data of the trotting Labrador retrievers and all CKCS was not included in a previous study.

The CKCSs had an mean age of 25 ± 15 months, an mean body weight of 7.9 ± 1.4 kg and a mean wither height of 32.8 ± 1.2 cm with a sex distribution of 2:1 (female: male). The Chihuahuas had an mean age of 38.5 ± 16.3 months, mean body weight of 2.8 ± 0.6 kg and an mean wither height of 19.9 ± 2.3 cm. The Labrador retrievers had an mean age of 21.3 ± 7.6 months, an mean body weight of 34.9 ± 5.5 kg and a mean wither height of 57.25 ± 4.2 cm. The sex distribution among the Chihuahuas and Labrador retrievers was 1:1.

### Ethics statement

This prospective study was conducted strictly according to the recommendations in the Guidelines for Care and Use of Laboratory Animals of the German Animal Protection Law. The protocol was approved by the Committee on the Ethics of Animal Experiments of the Justus-Liebig-University Giessen, Regierungspraesidium Hessen and Thuringia (Permit number: 22-2684-04-02-075/14). The study was conducted with the owners’ written informed consent.

### Study design

#### Clinical examination and cross-sectional imaging

All data were collected between 2015 and 2018 and analyzed thereafter. A complete general, neurological and orthopaedic examination was performed in all dogs. Neurological and orthopaedic examinations were performed by a board-certified veterinary specialist in neurology (ECVN). Magnetic resonance imaging (MRI) and computed tomography (CT) scans of the head and entire spine were acquired under general anaesthesia. A standard anaesthetic protocol was used (isoflurane inhalation narcotic 1.5–2.5%, premedication with diazepam 0.1 mg/kg and propofol 6 mg/kg). For CT, we used a 16-slice helical scanner (Brilliance Philips, 120kV, 200mAs, slice thickness 1mm) and bone and soft tissue windows were reconstructed. For MRI image acquisition before December 2016, a MRI 1.0 Tesla superconducting system Intera Philips, Netherlands, was used. For image acquisition after 2016, an MRI 3.0 Tesla Magnetom Verio Siemens, Germany, was used with a Syn-spine coil. T2-weighted images of the entire spine and the head were acquired in sagittal, dorsal and transverse planes. MRI and CT scans were performed with the dogs in dorsal recumbency and extended positioning of the CCJ. Based on the MRI images, CKCSs and Chihuahuas were graded for CLM according to the British Veterinary Association/Kennel Club Chiari malformation CM/SM classification criteria [24]. Only clinically sound dogs with CM 0/1 and SM 0 at the time of the investigation were included. Dogs with signs of atlantoaxial instability and/or overlapping [25] were not included. In all dogs, any abnormal findings in cross-sectional imaging led to exclusion from the study cohort.

#### Gait analysis and cineradiography on the treadmill

**Fig 1:**
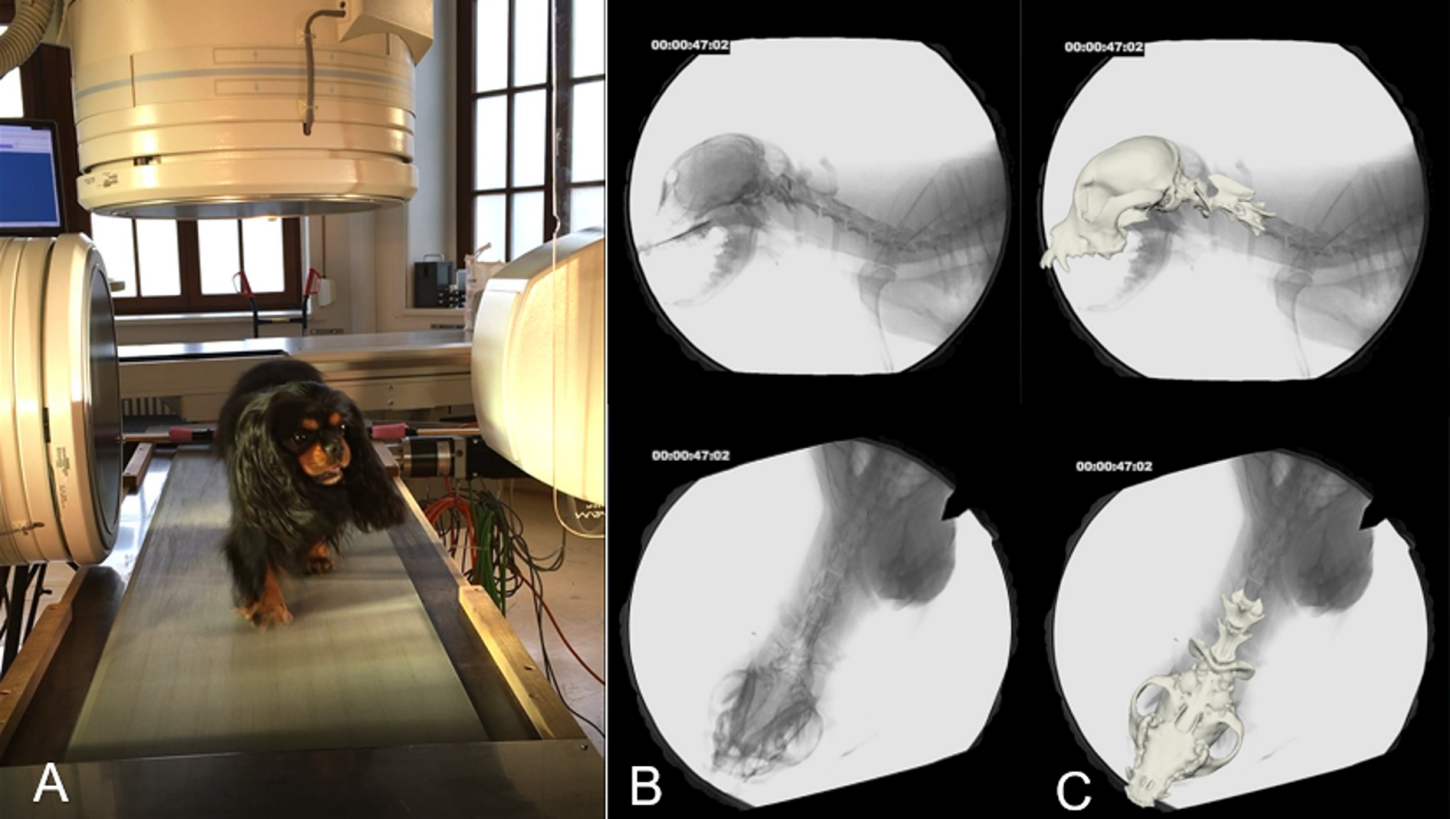
Biplanar fluoroscopy–setting and example. A: Biplanar fluoroscopy examination with a CKCS on the treadmill, 90° camera settings. B. Fluoroscopy images of the same CKCS in laterolateral and dorsoventral views. C. Fluoroscopy images and superimposed 3D bone model.

A horizontal motorised treadmill was used, and all dogs were habituated individually to their comfortable walking speed (S1 Table). A digital high-speed videography system (Neurostar Siemens) was used to record biplanar x-ray videos. Two high-speed cameras (Visario Speedcam, Weinberger) and two image intensifier systems with a diameter of 40 cm recorded the moving dogs synchronously from two perspectives (image resolution: 1.536 × 1.024 pixels; 500 frames per second), adjusted according to patient size. In CKCSs and Chihuahuas, a laterolateral and ventrodorsal projection (Fig 1), and in Labrador retrievers a left and right oblique projection with an angle of 63° was used. X-ray settings were 80–100 kV and 40–75 mAs with the shutter set at 500 μs. Synchronously recording standard high-speed video cameras (SpeedCam MiniVis, High Speed Vision) were used to determine the touchdown and lift-off events during the stride cycles and for general gait analysis. In most of the dogs, the time span between cross-sectional imaging and cineradiography was one to three months with a maximum time span of up to nine months.

#### Generation of a 3D bone model and Scientific Rotoscoping

Visualisation of bony elements and quantification of three-dimensional skeletal kinematics was achieved using Scientific Rotoscoping, a markerless and non-invasive technique of the X-Ray Reconstruction of Moving Morphology (XROMM) methodology [20].

**Fig 2:**
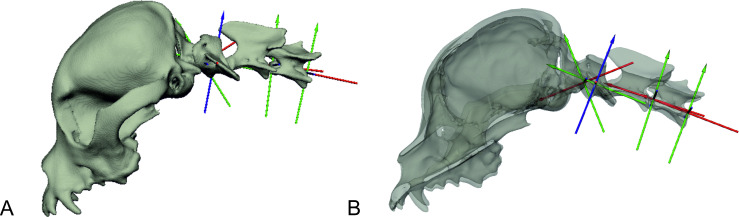
Cranial cervical spine and skull as bone models with virtual intervertebral joints. A: bone model of the skull and first three cervical vertebrae of a CKCS with coordinate axis of the virtual intervertebral joints; B: same bone model in a semi-transparent view.

A three-dimensional hierarchical bone model of the skull and first to third cervical vertebra (C1–C3) was generated with the image processing program Amira 6^®^ (Visage Imaging, Berlin, Germany) based on CT data sets. Virtual intervertebral joints (IVJ), based on previous radiological studies [26], were inserted into the bone model using the Autodesk Maya 2017^®^ software (Figs 1 and 2). The experimental setup was virtually reconstructed in Autodesk Maya^®^ and the bone model was matched to the biplanar fluoroscopy video frame by frame, resulting in a three-dimensional animation reflecting the *in vivo* movement of the dogs on the treadmill with high precision (S1-S10 Videos in S1 File). Temporal and spatial motion data were exported to Excel^®^. Each position of the individual elements of the bone model deviating from the starting position was automatically exported by the program in degrees or centimetres, allowing highly accurate motion analysis and measurements [22, 23, 27].

### Data analysis

**Fig 3:**
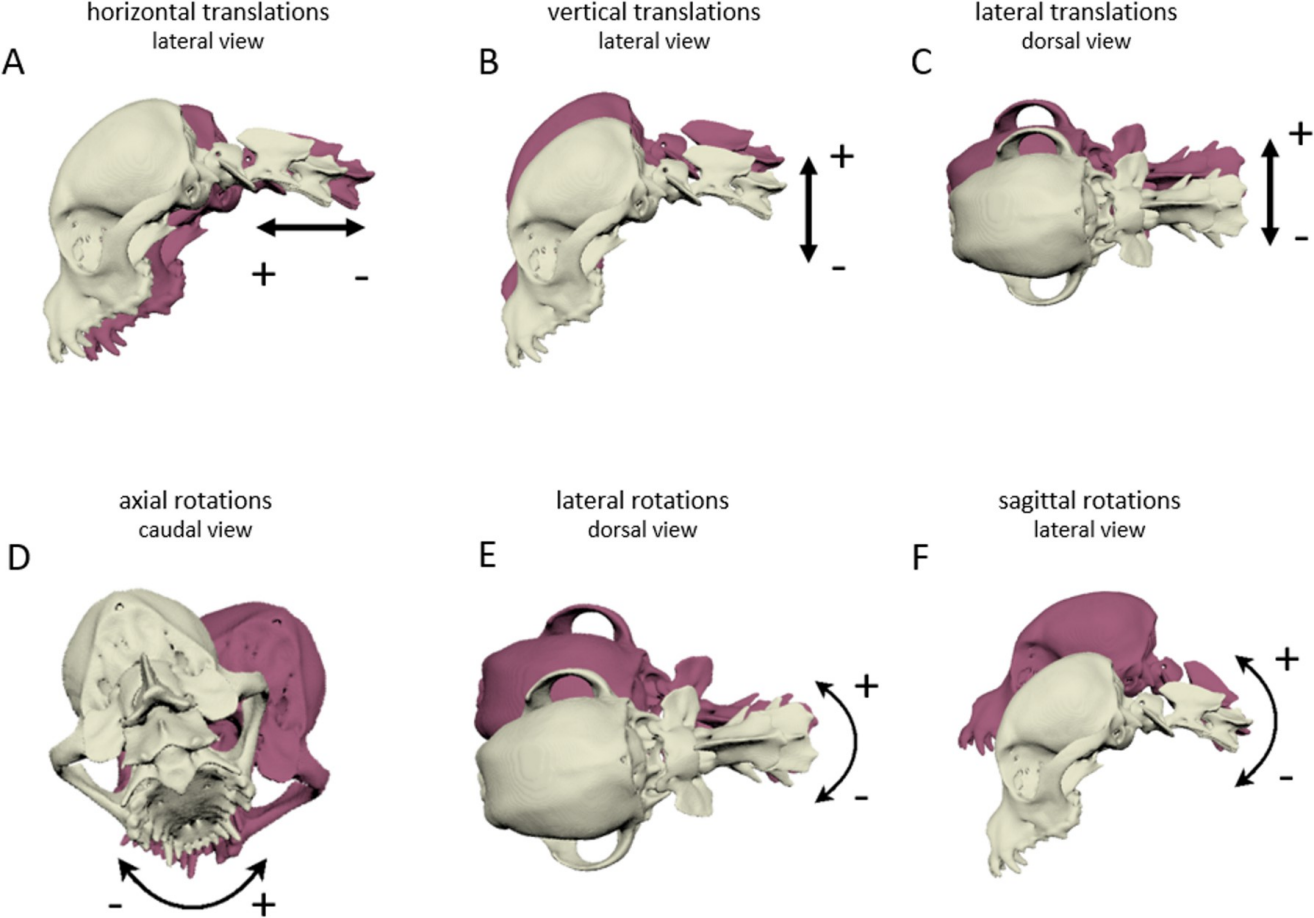
Translational and rotational movements in the bone models. Degrees of freedom during locomotion and arbitrary movements, demonstrated by XROMM images of the total cranial cervical spine. + indicates increasing values of the curves, − indicates decreasing values of the curves in Figs 5–7 in the direction of the arrows. A) horizontal translations along x-axis, lateral perspective, B) vertical translations along y-axis, lateral perspective, C) lateral translations along z-axis, dorsal perspective, D) axial rotations around x-axis, caudal perspective, E) lateral rotations around y-axis, dorsal perspective, F) sagittal rotations along z-axis, lateral perspective.

For all dogs examined, a time-normalisation (phase normalisation) for the swing and stance phase of each stride was executed based on the duty factor using Matlab^®^ software (The MathWorks, Inc., S1 Table) [28] to allow comparison of the angular movements across strides and dogs. Several trials were recorded and three to six consecutive strides for walk and trot were included. For further gait analysis, the stride length related to the dogs’ wither height was determined. Absolute motion measurements are depicted as the discrepancy of an actual position of a joint to the zero position, which is the positioning during CT examination. Translations of the joints are indicated in centimetres, rotations in degrees. Every curve deflection in the motion data of the virtual joints was correlated either to the stride cycle or to additional arbitrary movements of the head and neck observed in the high-speed videos. The 3D movements in six degrees of freedom (DOF) are defined as follows: axial rotations and horizontal translations occur along the craniocaudal axis of the body, lateral rotations and vertical translations along the ventrodorsal axis of the body and sagittal rotations as well as lateral translations occur along the laterolateral body axis (Fig 3). Translational movements of vertebra C3 as the uppermost hierarchical point of the bone model imitate the motion of the bone model as a whole (total cranial cervical spine, TCCS), including the dogs’ motion in space.

For all joints and DOF, the ROM and time of occurrence (TOO) were measured. The TOO represents the timing of minimal and maximal stride cycle-dependent curve deflections in one DOF and is expressed as the percentage of the stride cycle of the left hindlimb as a reference limb. For motion pattern recognition and ROM calculation, we focussed on steps in which the dogs kept their head in place, although arbitrary head movements could not be entirely eliminated. As stated before, the animation raw data of the Chihuahuas from Schikowski [23] were used. The ROM in the Chihuahuas had to be recalculated in order to provide a reasonable comparison between all dogs, because the method used for ROM calculation differs from Schikowski’s study. The ROM we calculated in this study represents the arithmetic means and standard deviations of the maximal magnitude of motion of a joint in one DOF during each complete stride cycle. Schikowski used an “average ROM” [23, 29], calculated from the arithmetic mean of each motion curve deflection during all stride cycles (S1 Fig). The Scientific Rotoscoping process of the Labrador retrievers and CKCSs in walk and trot, as well as all motion data calculations were obtained anew as mentioned above. For translational movements, a relative range of motion, which is the total range of motion correlated to the wither height, was calculated (example: total horizontal translation/wither height x 100). A comparison between the temporal and spatial motion patterns and motion magnitude of the CKCSs, Chihuahuas and Labrador retrievers was conducted.

**Fig 4:**
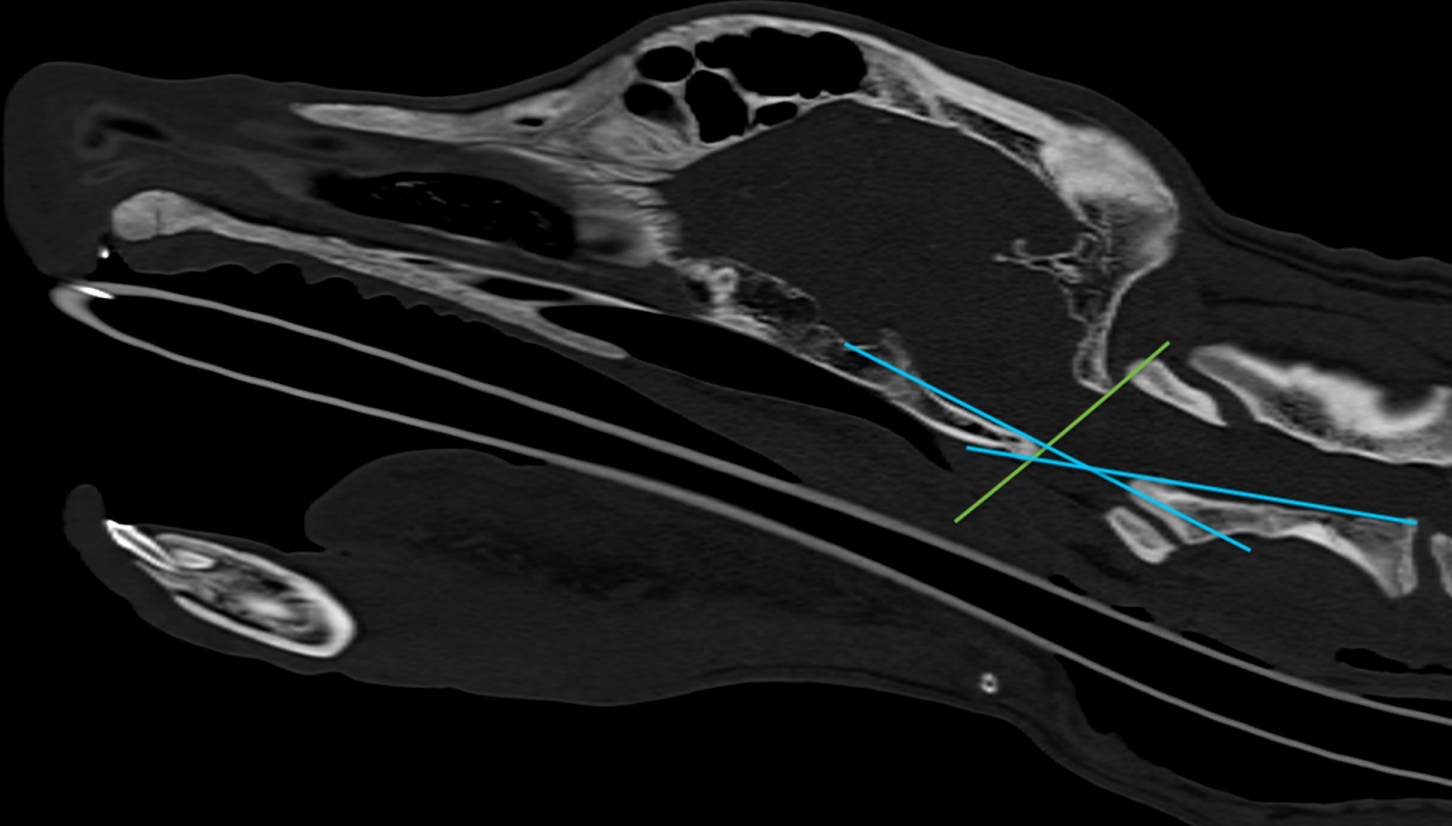
Analysis of physiologic relative position of the occipitoatlantoaxial joints. Sagittal CT image of the canine head with quantitative measurements as suggested by Waschk et al. [25]. McRae’s line (green): line drawn from basion to opisthion, no part of the dens or dorsal atlas arch is to cross this line. Wackenheim’s clivus baseline (rostral blue line): line drawn along the dorsal surface of the clivus, no more than one-third of the dens is to cross this line. Clivus canal angle (between both blue lines): angle formed by the junction of Wackenheim’s clivus baseline and the dorsal surface of the axis vertebral body.

The position of the CCJ in walking dogs was objectively described and quantified by using quantitative measurements of the CCJ position approved in human medicine and evaluated for veterinary medicine [25]. A McRae’s line and modified McRae’s line, Wackenheim’s clivus base line and clivus canal angle [25] were drawn into the fluoroscopic images in different head–neck positions of one breed representative each (Fig 4).

Additionally, a personal measurement error and interobserver variability was calculated. The personal measurement error was calculated from the motion data of the CKCSs and Labrador retrievers in trot. One complete stride cycle of one Labrador retriever (L3) and one CKCS (CKCS 10) was animated three times immediately one after another. One complete stride cycle of another Labrador retriever (L4) and another CKCS (CKCS 2) were animated three times at intervals of approximately 12 hours. These animations were compared at five fixed times during the stride cycle and the absolute differences in position of each animated bone (in centimetres or degrees) between the three trials were calculated. The arithmetic mean and standard deviation between the three trials were defined and delivered the personal measurement error.

The interobserver variability of the bone model animations of the authors MN and LS [23] was assessed by calculating the average difference between the motion curves of both authors animating three Labrador retrievers (L1-3) in walk in three to six consecutive strides. The total difference between the motion curves in centimetres for translational movements and in degrees for rotational movements and the according mean values and standard deviations were calculated for every percentage point of each stride. Previously, the difference between the initial starting points of the curves of both authors was subtracted from MN’s motion data to rule out variations due to technically conditioned different zero positions. The percentage of the calculated deviation of the results of both authors from the ROM of each joint and DOF was determined.

### Statistical analysis

A test for normal distribution of the ROM of the atlantoaxial and atlantooccipital joints was performed using the Shapiro–Wilk test. To identify ROM differences and differences in the average sagittal rotation of the atlas between the breed groups, we performed a one-way ANOVA in case of normal distribution of the data or a Kruskal-Wallis test, when normal distribution of the data was not confirmed. In case of significant differences between the groups, a post-hoc test (Dunn-Bonferroni test) was calculated. ROM differences between walk and trot were calculated using a two-tailed t-test in case of normal distribution of the data or otherwise a Mann–Whitney U-test was performed. P-values less than 0.05 were considered statistically significant. For selected movements of the IVJs, Spearman’s rank correlation coefficients were calculated between the breed groups.

## Results

### Comparison of step length

The step length in relation to wither height was largest in CKCSs in walk and trot (step length: wither height in walk: CKCS 1.32 ± 0.14; Chihuahua 1.17 ± 0.21; Labrador 1.27 ± 0.19, and in trot: CKCS 1.88 ± 0.13; Chihuahua 1.34 ± 0.32; Labrador 1.77 ± 0.08). The difference in step length was significant only for CKCSs and Chihuahuas in walk (p-value < 0.05).

### Translational movements of the total cranial cervical spine (TCCS)

**Fig 5:**
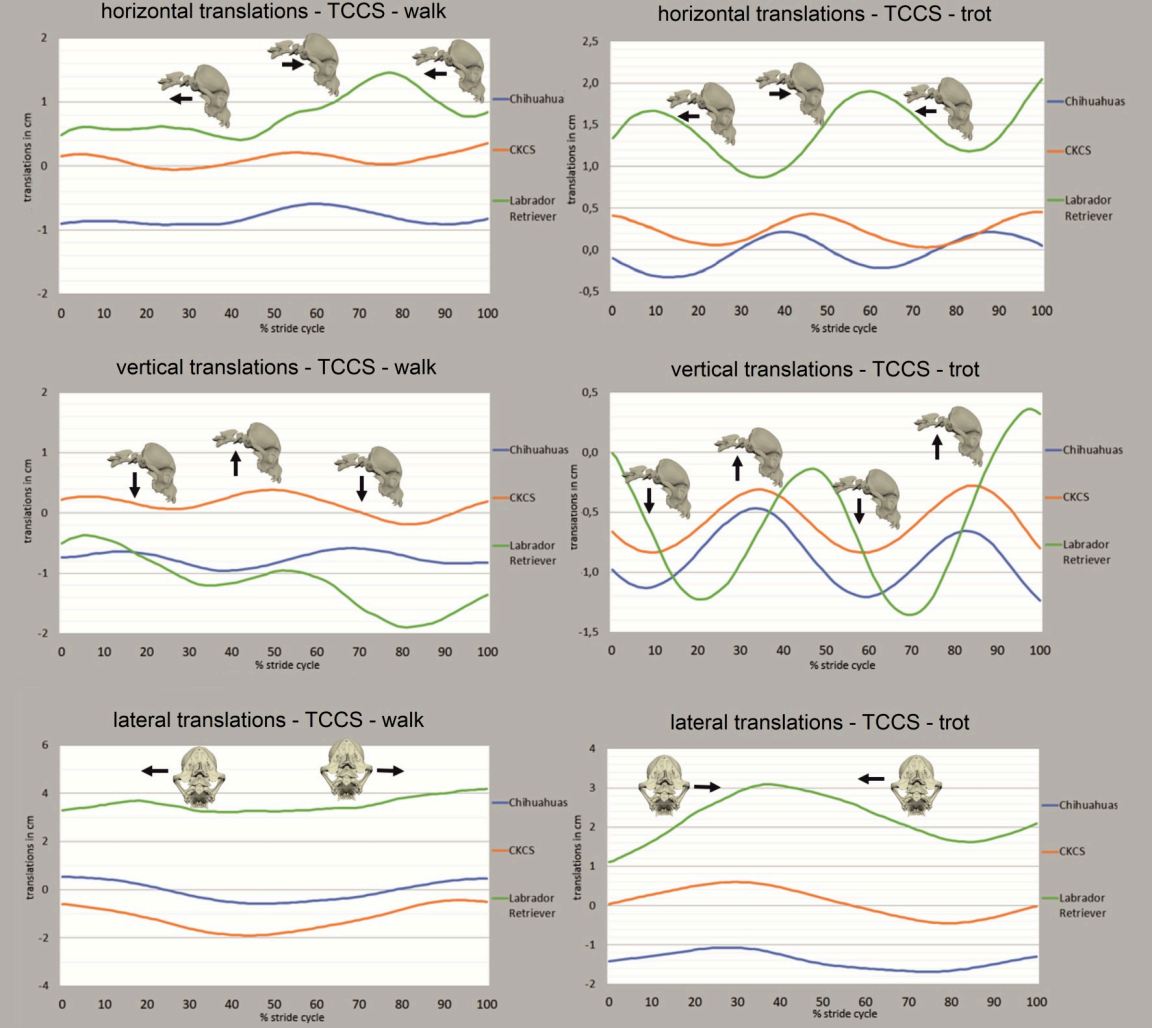
Phase-normalised translational movements of the total cranial cervical spine (TCCS) in walk and trot. All translational degrees of freedom as mean values for all strides of Chihuahuas (blue), CKCSs (orange) and Labrador retrievers (green) are depicted. X-axis represents one complete stride cycle from touchdown (0%) to subsequent touchdown (100%) of the left hind limb. Y-axis represents the motion amplitude in cm. Bone models and arrows indicate direction of stride cycle-dependent bone movement.

The same basic motion patterns of the TCCS were recognised in all breeds and all DOF. **Horizontal translation** consistently showed a biphasic motion pattern in walk and trot (Fig 5). In a biphasic motion pattern, two maximum and two minimum motion deflections are encountered during one complete stride cycle. Maximum values were correlated to the late stance phase and lift-off events of the pelvic limbs with a prolonged advance in the Labrador retrievers, especially in trot (S2 Table). Absolute and relative ROM are found in Table 1.

**Table 1:** Range of motion of the translations and rotations of the total cranial cervical spine (TCCS).

Breed	DOF	Walk		Trot	
		absolute ROM	relative ROM	absolute ROM	relative ROM
CKCS	horizontal translation	2.03 ±1.28	6.10 ± 3.93	1.53 ± 0.39	4.74 ± 1.24
Labrador		2.31 ± 0.13	3.91 ± 0.35	1.94 ± 0.07	3.41 ± 0.24
Chihuahua		1.34 ± 0.83	6.02 ± 3.94	1.28 ± 0.31	6.04 ± 1.79
CKCS	vertical translation	1.82 ± 0.42	5.46 ± 1.27	1.69 ± 0.43	5.21 ± 1,31
Labrador		2.65 ± 0.83	4.53 ± 1.56	2.17 ± 0.24	3.78 ± 0.1
Chihuahua		1.56 ± 0.79	7.10 ± 4.01	1.45 ± 0.38	6.70 ± 1.58
CKCS	lateral translation	2.63 ± 0.72	7.89 ± 2.25	1.72 ± 0.40	5.32 ± 1.19
Labrador		3.36 ± 1.66	5.71 ± 2.93	2.67 ± 0.03	4.70 ± 0.43
Chihuahua		2.07 ± 0.50	9.16 ± 2.37	1.00 ± 0.27	4.75 ± 1.55
CKCS	Axial rotation	6.51 ± 2.97		6.87 ± 4.10	
Labrador		12.07 ± 1.29		7.49 ± 1.35	
Chihuahua		8.95 ± 1.24		7.05 ± 2.30	
CKCS	lateral rotation	7.81 ± 2.97		6.53 ± 2.17	
Labrador		12.25 ± 1.73		8.18 ± 2.15	
Chihuahua		8.40 ± 2.51		5.40 ± 1.65	
CKCS	sagittal rotation	9.01 ± 4.04		8.87 ± 2.97	
Labrador		12.58 ± 2.71		7.94 ± 1.80	
Chihuahua		12.06 ± 3.45		7.28 ± 2.46	

Means and standard deviations averaged for all CKCSs, Labrador retrievers and Chihuahuas in walk and trot. Absolute range of motion indicates the measured range of motion in cm or °, relative range of motion for the translations indicates the absolute range of motion related to the wither height of the dogs in % [(absolute ROM in cm/ wither height in cm) x 100].

**Vertical translation** of the TCCS showed a biphasic motion pattern with dorsal translations in the last third of the swing phase and ventral translation in the first third of the stance phase of the front limbs (Fig 5). In trot, minimum values coincided with the mid-stance phase of the front limbs, while dorsal translation of the TCCS was related to the lift-off events of the front limbs (S2 Table). Absolute and relative ROM are depicted in Table 1.

**Lateral translation** of the TCCS in walk was characterised by a monophasic motion pattern in all breeds (Fig 5). In a monophasic motion pattern, only one maximum and one minimum motion deflection during a stride cycle occur. Maximum lateral translations to the left and right at the beginning of the stance phase of the ipsilateral front limb were detected in all breeds in walk. The CKCSs and Labrador retrievers showed delayed maximum and minimum values in trot during the second half of the front limb stance phase (TOO: S2 Table, ROM: Table 1).

Comparing the translational ROM normalised to body size, the greatest values were recognised in the Chihuahuas except of lateral translation in trot. In trot, the ROM was smaller in all translational DOF compared to walk in all breeds (Table 1). In general, changes of the dogs’ position on the treadmill affected the curve position on the y-axis for translational movements.

### Rotational movements of the TCCS

**Fig 6:**
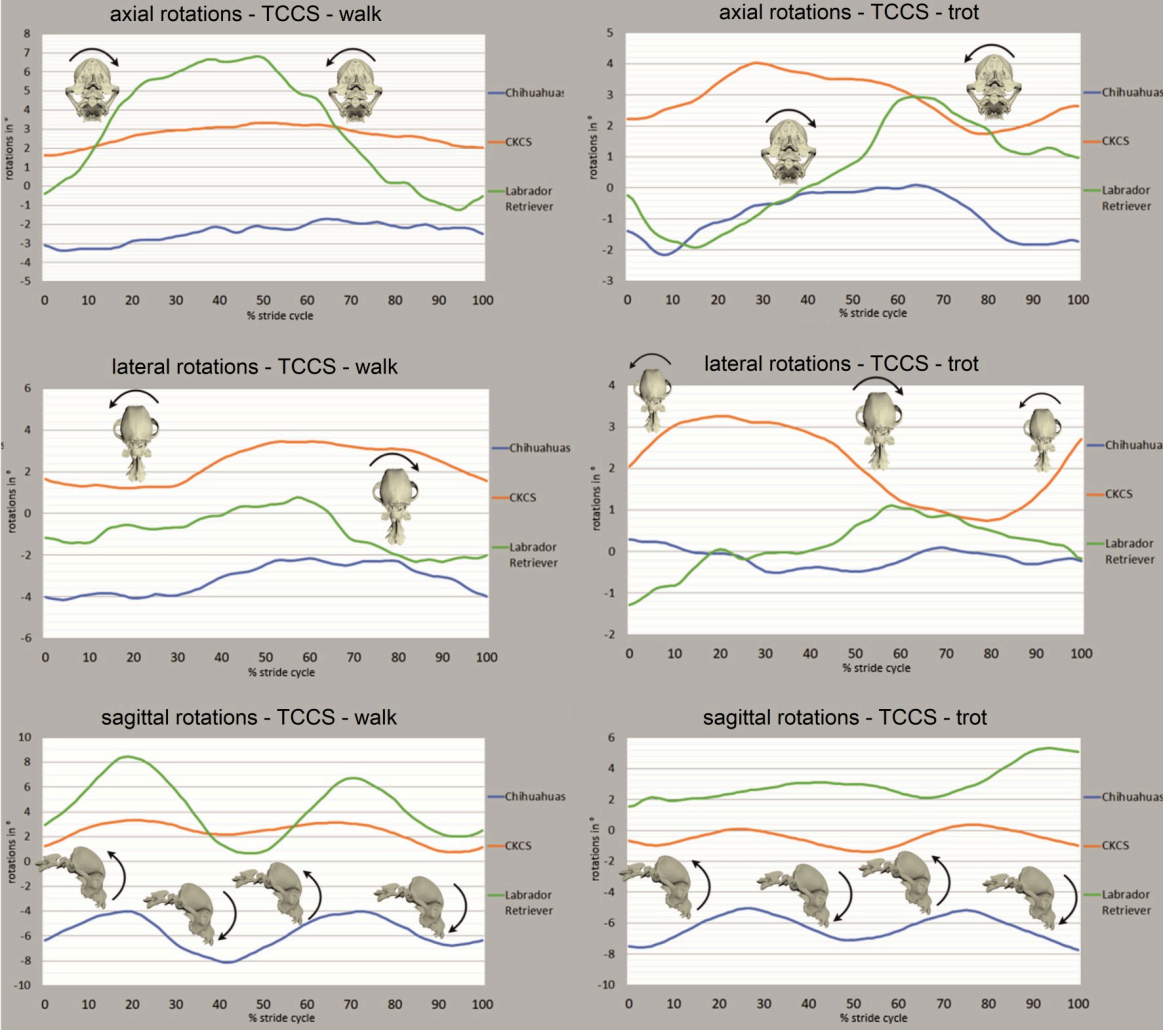
Phase-normalised rotational movements of the total cranial cervical spine (TCCS) in walk and trot. All rotational degrees of freedom as mean values for all strides of Chihuahuas (blue), CKCSs (orange) and Labrador retrievers (green) are depicted. X-axis represents one complete stride cycle from touchdown (0%) to subsequent touchdown (100%) of the left hind limb. Y-axis represents the motion amplitude in degrees. Bone models and arrows indicate direction of stride cycle-dependent bone movement.

Rotational movements were strongly affected by both gait cycle-dependent and arbitrary movements of the head and neck. For **axial rotation** of the TCCS in walk, a stride cycle-dependence was recognised in all Labrador retrievers and inconsistently in the CKCSs and Chihuahuas (Fig 6). The axial rotation was characterised by a monophasic motion pattern with an axial rotation towards the ipsilateral front limb during its stance phase. In trot, a uniform monophasic motion pattern was recognised in seven of eight CKCSs and one Chihuahua and Labrador retriever each.

The stride cycle-dependent **lateral rotation** was characterised by a monophasic motion pattern, inconsistently observed in all breeds (Fig 6). In walk, dogs of all breeds investigated showed lateral rotation of the TCCS to the right or left during the first third of the stance phase of the front limbs but no uniform correlation to the footfall pattern was observed. (S2 Table). In trot, a stride cycle-dependent lateral rotation of the TCCS was found in the CKCS. A left lateral rotation was correlated to the second half of the stance phase of the right front limb. In the Labrador retrievers, the time and direction of the lateral rotation were less consistent with various results for all dogs. The stride cycle-dependent pattern in trot was not observed in the Chihuahuas. The ROM of lateral rotations of the TCCS is depicted in Table 1. Axial and lateral rotation appeared to be positively correlated with each other, implying a simultaneous, but oppositely directed rotation (Spearman’s rank correlation coefficient 0.51 in walk, 0.49 in trot, result not statistically significant).

**Sagittal rotation** of the TCCS was characterised by a biphasic motion pattern in all breeds in walk and trot (Fig 6). In most of the dogs, a dorsal sagittal rotation (extension) was related to the second half of the swing phase of the front limbs with maxima related to the touchdown events in walk. Ventral rotation (flexion) was associated with the first half of the stance phase of the front limbs. ROM of sagittal rotations are depicted in Table 1.

The total range of motion of the rotational DOF of the TCCS in all breeds investigated was greater in walk than in trot (except axial rotation in CKCSs, Table 1). Sagittal rotation of the TCCS showed a medium positive correlation with vertical translation (Spearman’s rank correlation coefficient 0.43 in walk, 0.44 in trot; result not statistically significant).

During Scientific Rotoscoping, no rotational movements of IVJ C2/C3 above the resolution limits of the technique [30] were detected. The vertebrae C2 and C3 remained in unchanged position in all breeds in walk and trot, therefore no results on the IVJ C2/C3 can be reported.

### Rotational movements of the atlantoaxial joint

**Fig 7:**
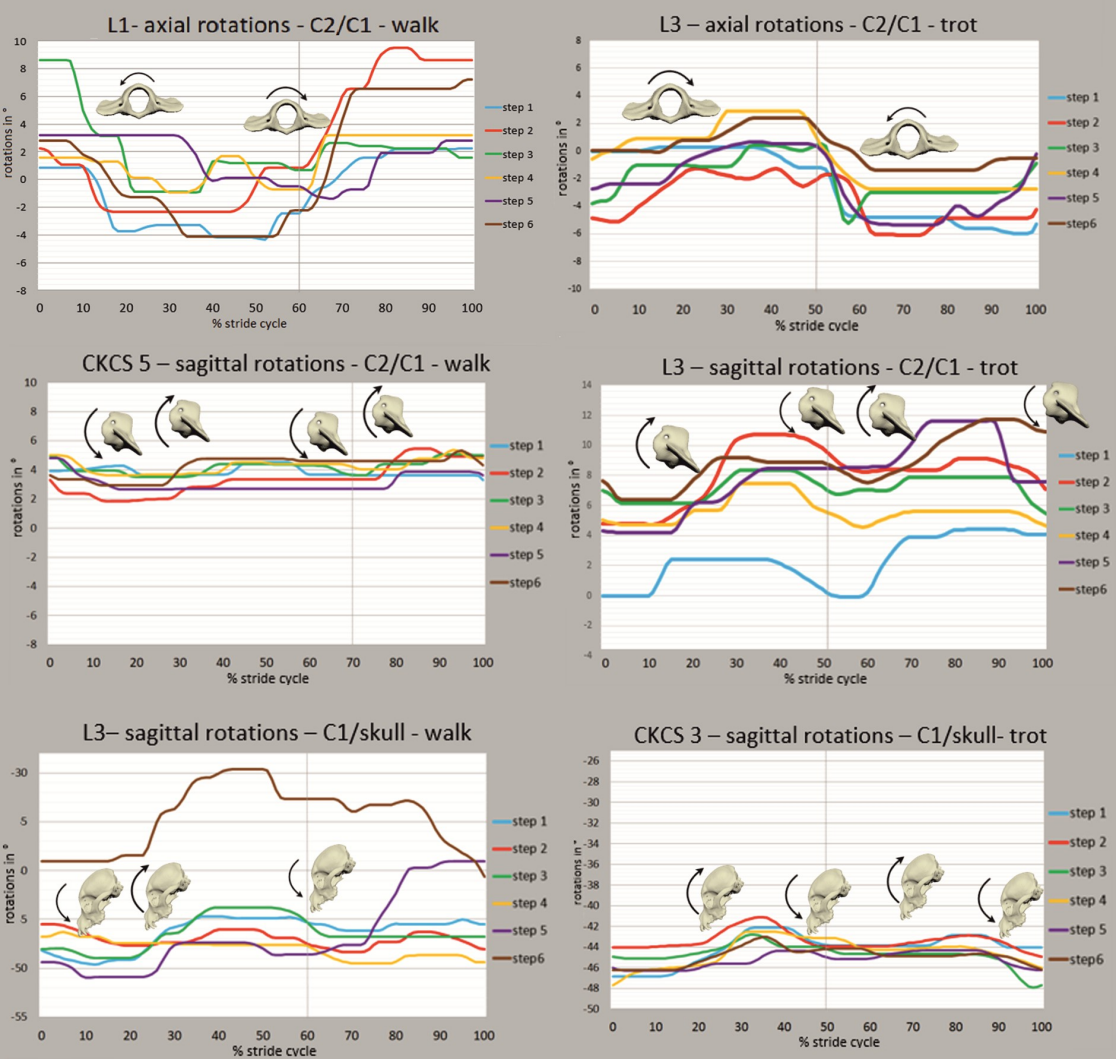
Examples of phase-normalised movements of the atlantoaxial (C2/C1) and atlantooccipital (C1/skull) joint in walk and trot. All stride cycle-dependent rotational degrees of freedom in CKCSs and Labrador retrievers in six strides. X-axis represents one complete stride cycle from touchdown (0%) to subsequent touchdown (100%) of the left hind limb. Y-axis represents the motion amplitude in °. The vertical line represents the duty factor. Bone models and arrows indicate direction of stride cycle-dependent bone movement.

**Axial rotation** of the atlantoaxial joint was observed as a monophasic stride cycle-dependent pattern in all Labrador retrievers, two CKCSs and one Chihuahua (Fig 7). In trot, a stride cycle-dependence of axial rotation was first described in this study in two of three Labrador retrievers and two of eight CKCSs. When correlated to the stride cycle, an axial rotation to the right side was associated with the second half of the left front limb’s stance phase with a directional change in the last third of the stance phase and corresponding results for left axial rotation (S3 Table). ROM of axial rotations are depicted in Table 2. Although not statistically significant, axial rotation of the TCCS was correlated to axial rotation of the atlantoaxial joint to the opposite direction in walk in some dogs (Spearman’s rank correlation coefficient −0.43 in Labrador retrievers). In trot, simultaneous and unidirectional axial rotation of the TCCS and atlantoaxial joint were observable in most CKCSs, but without statistical significance.

**Table 2:** Range of motion of the rotations of C2/C1 IVJ (atlantoaxial joint).

Breed	DOF	Walk	Trot
CKCS	sagittal rotation	3.64 ± 1.05	2.62 ± 0.65
Labrador		2.50 ± 0.28	3.35 ± 1.37
Chihuahua		5.34 ± 1.84	0.87 ± 0.74
CKCS	axial rotation	3.83 ± 1.05	3.18 ± 2.75
Labrador		7.81 ± 2.01	4.06 ± 2.13
Chihuahua		10.69 ± 2.96	6.06 ± 2.24
CKCS	lateral rotation	5.99 ± 1.91	4.60 ± 1.49
Labrador		5.18 ± 0.50	3.31 ± 1.41
Chihuahua		7.85 ± 1.55	2.93 ± 0.98

ROM in °, means and standard deviations averaged for all CKCSs, Labrador retrievers and Chihuahuas in walk and trot.

For **sagittal rotation** of the atlantoaxial joint, there were only very inconsistent, partly stride cycle-dependent motions visible (Fig 7). Most curve deflections were caused by the dogs’ arbitrary head movements. A sagittal rotation of the atlantoaxial joint in opposite direction to the sagittal rotation of the TCCS could be observed simultaneously in a few steps of individual dogs of all breeds. ROM of sagittal rotations are depicted in Table 2.

The mean sagittal rotation of the atlantoaxial joint during all stride cycles investigated was greatest in the Chihuahuas in walk and trot. Labrador retrievers and CKCSs showed markedly smaller values, around half of those of the Chihuahuas (Table 3). These differences were not statistically significant (S5 Table). An extension of the neck to an upright position and flexion of the atlantooccipital joint was associated with a positive sagittal rotation of the atlantoaxial joint. Spearman’s rank correlation coefficients for these sagittal rotations were weak (−0.2–0.1) and not statistically significant.

**Table 3:** Mean ± standard deviation of mean sagittal rotation of C2/C1 IVJ (atlantoaxial joint).

Breed	Mean sagittal rotation	
	**Walk**	**Trot**
CKCS	6.93 ± 4.62	7.25 ± 6.7
Labrador	9.23 ± 2.75	6.28 ± 3.15
Chihuahua	13.46 ± 7.39	14.17 ± 4.77

Mean sagittal rotation in °, for all strides in all CKCSs, Labrador retrievers and Chihuahuas in walk and trot.

In none of the breeds, **lateral rotation** of the atlantoaxial joint could be correlated with the stride cycle but was detected in arbitrary head movements to the sides. ROM are depicted in Table 2. Again, in all breeds, the ROM of lateral rotation was greater in walk than in trot (Table 2). Lateral rotation was the predominant DOF in CKCSs in walk and trot, while in Chihuahuas and Labrador retrievers, axial rotation exhibited the largest ROM.

The ROM of the atlantoaxial joint showed normal distribution in walk and trot among the breeds in all DOF (p-value > 0.05) (S6 Table). There were no significant differences in the ROM among the breeds, neither in walk nor in trot, in all rotational DOF of the atlantoaxial joint (S7 Table). The differences in ROM in walk and trot were significant for the atlantoaxial joint in all breeds (S8 Table).

### Rotational movements of the atlantooccipital joint

**Sagittal rotation** showed only inconsistent stride cycle-dependent curve deflections in walk and trot in single individuals of all breeds (Fig 7, S4 Table). If not superimposed by arbitrary head movements, sagittal rotation of the atlantooccipital joint was observed simultaneously but oppositely directed to the sagittal rotation of the TCCS. ROM are depicted in Table 4. Sagittal rotation was the main rotational DOF of the atlantooccipital joint in all dog breeds included. The mean sagittal position for the atlantooccipital joint, in respect to the extended zero position during the CT examination, showed negative total values. There was a medium negative correlation between sagittal rotation of the TCCS and atlantooccipital joint in the CKCSs in trot (−0.41), but without statistical significance. Other Spearman’s rank correlation coefficients for sagittal rotations were weak.

**Table 4:** Range of motion of the rotations of C1/skull IVJ (atlantooccipital joint).

Breed	DOF	Walk	Trot
CKCS	sagittal rotation	5.75 ± 2.36	5.25 ± 1.98
Labrador		6.94 ± 0.48	5.52 ± 0.96
Chihuahua		10.95 ± 3.73	6.45 ± 2.49
CKCS	axial rotation	1.80 ± 2.32	3.10 ± 2.00
Labrador		8.47 ± 2.03	1.38 ± 0.39
Chihuahua		1.75 ± 0.97	6.81 ± 3.11
CKCS	lateral rotation	4.20 ± 2.32	3.69 ± 1.87
Labrador		3.77 ± 0.72	3.34 ± 0.32
Chihuahua		6.54 ± 1.85	4.09 ± 1.20

ROM in °, means and standard deviations averaged for all CKCSs, Labrador retrievers and Chihuahuas in walk and trot.

**Axial and lateral rotations** of the atlantooccipital joint were not correlated with the stride cycle, but rather reflected arbitrary head movements. Both occurred simultaneously as a coupled motion pattern, mainly directed to the same side. ROM are depicted in Table 4.

There were no significant differences in the ROM of the atlantooccipital joint among the breeds, neither in walk nor in trot, in all rotational DOF, except a smaller axial rotation in Chihuahuas in trot (S6 and S7 Tables). The differences in ROM in walk and trot were not significant for the atlantooccipital joint (S8 Table).

### Description of CCJ and skull position

The posture of the neck during walking and trotting differs individually in each dog. Interestingly, a relative alignment of the neck to the level of the thoracolumbar spine was observed in most CKCSs (5 of 8) and the Labrador retrievers (3 of 3) in walk. Most of the Chihuahuas (5 of 8) showed a more upright posture of the neck, carried above the level of the withers. Nevertheless, a wide range of flexion and extension of the head and neck were observed associated with arbitrary head movements in all breeds. Regardless of the breed, we observed a positive sagittal rotation (extension) of the atlantoaxial joint when the neck was raised either during locomotion or arbitrary head movements, which brought the atlas to a steeper angle. This was reflected in the data of mean sagittal rotation of the atlantoaxial joint, where highest values were calculated in the Chihuahuas (Table 3). Associated with this, a coupled negative sagittal rotation (flexion) of the atlantooccipital joint was observed, especially when the gaze of the dog remained directed forward. Nevertheless, sagittal rotation of the atlantooccipital joint in walk in the Chihuahua group was not significantly different compared to other dog breeds (CKCS −35.94 ± 15.76°; Chihuahua −33.71° ± 14.15°; Labrador −41.10 ± 2.87°).

**Fig 8:**
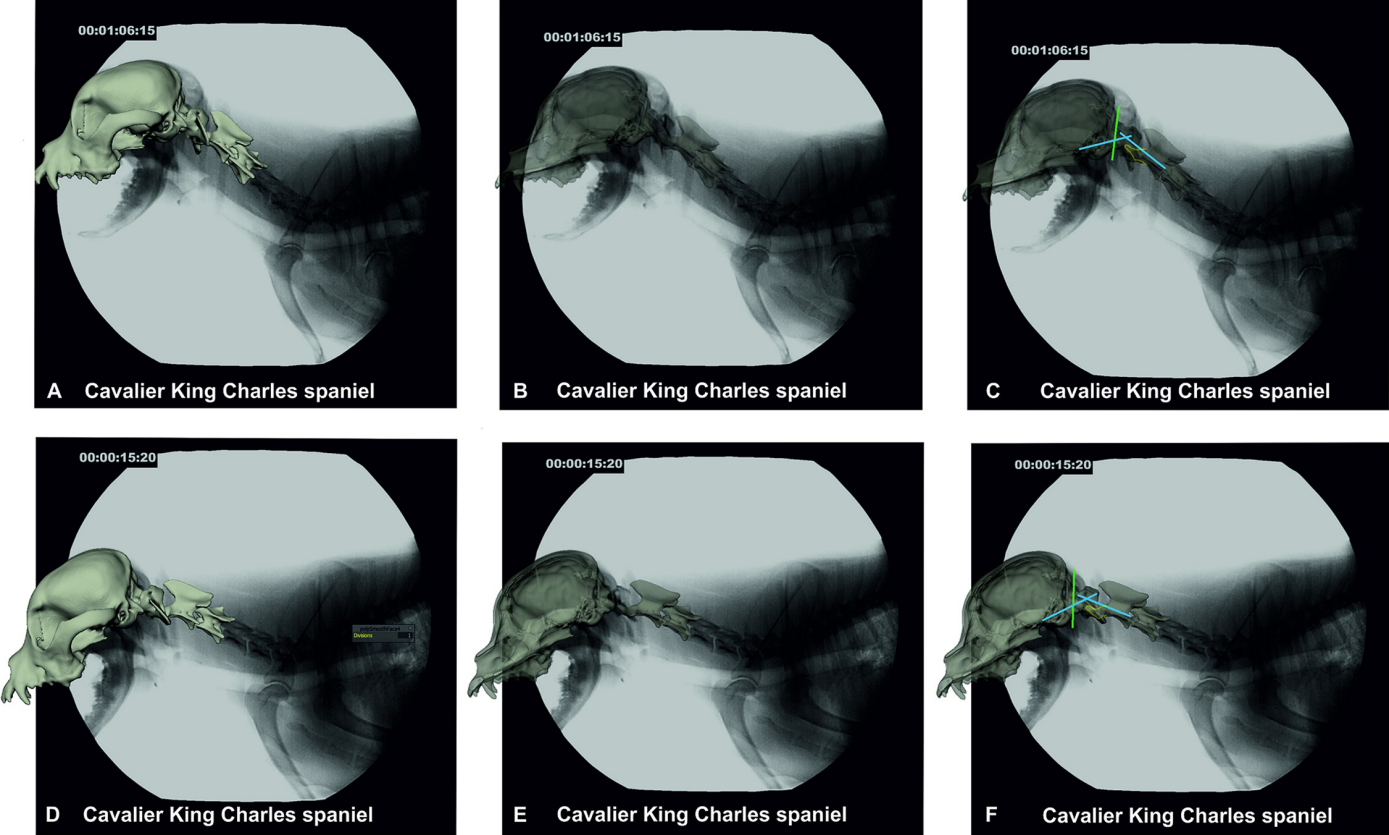
CCJ position in a walking CKCS. Images A–C: CKCS 2 in walk with an elevated neck position. Images D–F: CKCS 2 in walk with a lowered neck position. A/D: fluoroscopy image with superimposed bone model, B/E: semi-transparent bone model and transparent atlas, C/F: semi-transparent bone model and illustration of the (modified) McRae’s line (green line). Wackenheim’s clivus baseline (rostral blue line) and clivus canal angle (both blue lines, angle in image C 127°, image F: 131°). The contour of the dens is highlighted in yellow. 90° camera position.

**Fig 9:**
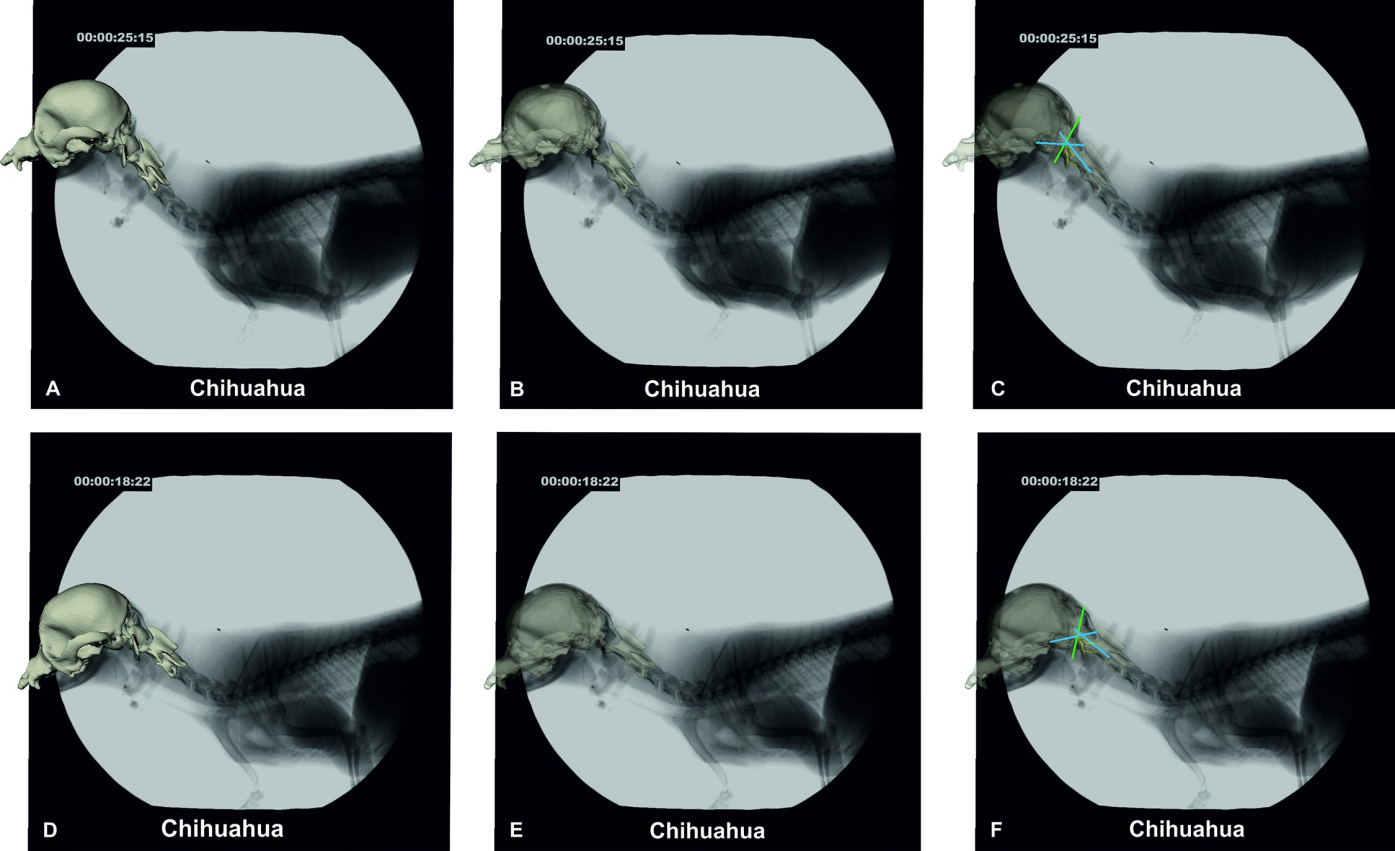
CCJ position in a walking Chihuahua. Images A–C: Ch6 in walk with an elevated neck position. Images D–F: Ch6 in walk with a lowered neck position. A/D: fluoroscopy image with superimposed bone model, B/E: semi-transparent bone model and transparent atlas, C/F: semi-transparent bone model and illustration of the (modified) McRae’s line (green line), Wackenheim’s clivus baseline (rostral blue line) and clivus canal angle (both blue lines, angle in image C and F: 129°). The contour of the dens is highlighted in yellow. 90° camera position.

**Fig 10:**
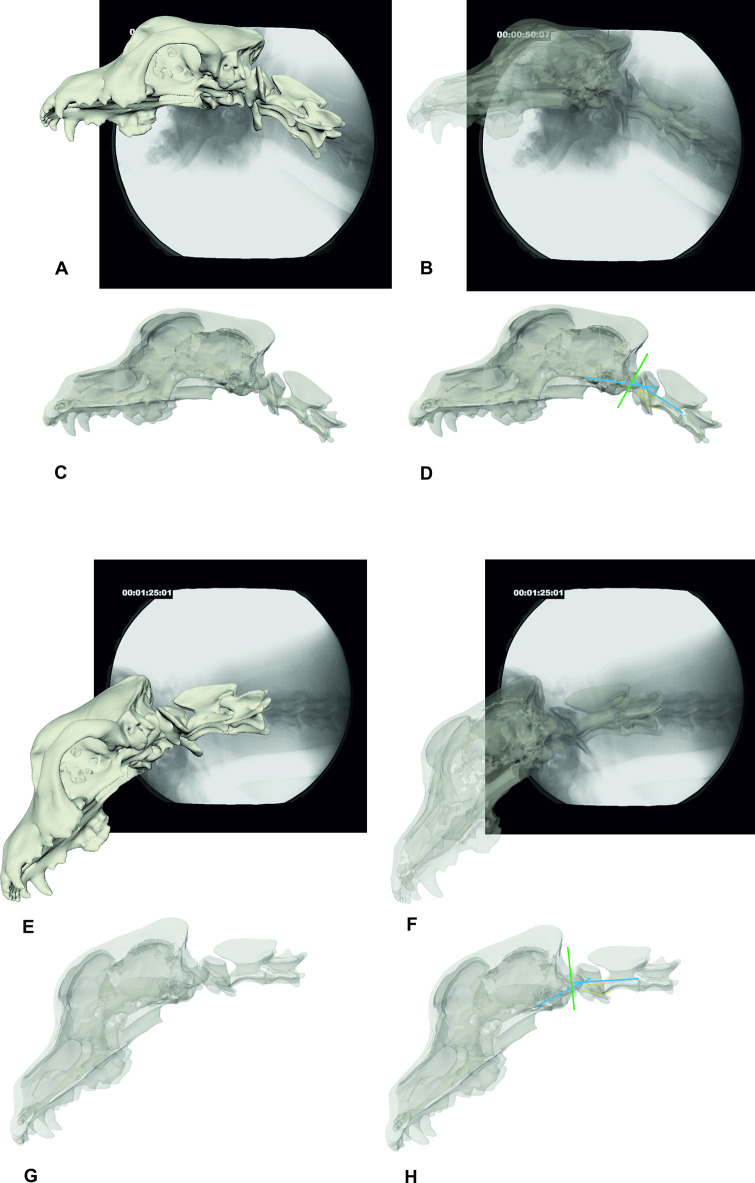
CCJ position in a walking Labrador retriever. Images A–D: L1 in walk with an elevated neck position, E–H lowered neck position. A/E: fluoroscopy image with superimposed bone model, 63° oblique camera position. B/F: semi-transparent bone model and transparent atlas, 63° oblique camera position. C/G: semi-transparent bone model, laterolateral view, D/H: semi-transparent bone model in laterolateral view with illustration of the (modified) McRae’s line (green line), Wackenheim’s clivus baseline (rostral blue line) and clivus canal angle (both blue lines, angle in image D: 154°, in image H 153°). The contour of the dens is highlighted in yellow.

In none of the dogs, neither the dens nor the dorsal atlas arch crossed McRae’s line (Figs 8–10). In none of the dogs, more than one-third of the dens did cross Wackenheim’s clivus baseline. The clivus canal angle was subjectively highly variable depending on the head and neck position of the dog with values ranging between 128–154° in three breed representatives. The clivus canal angles of the dogs are illustrated in Figs 8–10. These quantitative measurements used by Waschk et al. [25] are suggested to characterise the physiologic position of the CCJ in tomographic imaging studies in dogs under general anaesthesia.

### Measurement error and interobserver correlation

A personal measurement error was calculated for the motion data of CKCSs and Labrador retrievers in trot. An error of 0.01 cm in CKCSs in a 90° camera position and 0.04 cm in Labrador retrievers in a 63° oblique camera position in translational movements was calculated. For rotational movements, the personal measurement error was 0.32° in CKCSs and 0.4° in Labrador retrievers. The mean value of the difference between the motion data curves of the authors MN and LS (interobserver variability) was smallest for translations and rotations of the TCCS (5.7 ± 1.0% of the ROM averaged for all translational DOF, 9.9 ± 2.3% of the ROM averaged for all rotational DOF, S9 Table). For all rotational DOF of the atlantoaxial joint, the mean deviation from the ROM calculated was 21.7 ± 3.1% and 24.8 ± 6.5% for the atlantooccipital joint (S9 Table). Large deviations between the observers’ animations were calculated for axial rotations of the atlantooccipital joint (31.7% of ROM).

## Discussion

CKCSs and Chihuahuas are, amongst other breeds, predisposed to morphological alterations of the cranial base [31] and CCJ [7, 32]. While the morphological aberrations of the skull and cervical spine are well described [7, 8, 33–35], their potential implications for pathological movements and instability are not well characterised. Assessment of instability is usually limited to static imaging methods [25, 36–38]. The relative position of the CCJ of dogs under general anaesthesia is likely to differ from a dog in the standing position, and even more so in motion [39, 40]. In this study, we examined the kinematics of the CCJ and cranial cervical spine of clinically sound CKCSs, Chihuahuas and Labrador retrievers using Scientific Rotoscoping. The capabilities of the the method in visualising and measuring detailed three-dimensional skeletal kinematics in dogs have been demonstrated in multiple previous studies [21, 41, 42]. Scientific Rotoscoping is a very time-consuming method and this study to date provides the largest amount of data collected with Scientific Rotoscoping on CCJ motion in dogs during locomotion. Stride cycle-dependent motion patterns and ROM of the CCJ were the focus of our work, as this reflects the impact of recurrent forces acting on the cervical spine during locomotion, knowledge of which may be of interest for CCJA treatment and post-surgical CCJ biomechanics as well as implant stability. In this study, we gave the first detailed description of the movement of the CCJ in trot in three different dog breeds. A lateral rotation of the canine TCCS in trot could be related to the stride cycle for the first time, as it was described by Schikowski [23] for walk. We found that the basic motion patterns of the TCCS are similar in walk and trot with minor individual and temporal differences of maxima and minima. Although the footfall pattern of walk and trot differ, the basic cervical and craniocervical motion patterns are conserved with more or less distinctness. Sagittal and axial rotations of the atlantoaxial and atlantooccipital joint are generally detectable simultaneously, but in opposite directions to the movement of the TCCS. These gait-dependent motions seem to display mostly compensatory movements in relation to the gross movement of the cervical spine in order to provide a stable head position.

Gait cycle-dependent motion patterns of the TCCS and CCJ were not significantly different between the breeds included, although there was mild individual temporal and spatial variation. This is most likely due to individual gait-specific factors and physical properties like body mass or length. The gross species-specific anatomy and head–neck posture in moving dogs as well as basic muscle activities seem to outrange breed-specific morphological characteristics.- Nevertheless, this result has to be tempered by a still relatively small study population. Multiple anatomical differences of the occiput, atlas and axis, like larger spheno-occipital angulation or the reduced craniocaudal and increased dorsoventral diameter of the atlas [6, 43–46] are described in small breed and brachycephalic dogs. Presumably, these morphological variances could shift the centre of rotation of the CCJ joints cranially and induce differences in motion patterns and possible ranges of motion, but this was not detectable in our study. Comparative studies concerning the centres of rotation of the CCJ in different dog breeds are missing. At least, Penning and Badoux [26] could not detect different centres of rotation in the cranial cervical spine in large and medium breed dogs.

In this study, we decided on calculating the ROM differently compared to our previous study [23]. The average ROM used by Schikowski [23] can easily be affected by very small and potentially user dependent curve deflections during the Rotoscoping process, especially when only minimal rotations occur (S1 Fig). Therefore, a maximum ROM per stride cycle averaged for all strides seemed to be more reasonable. Calculations for the Chihuahuas, as mentioned before, were adapted to this method and explain the differences in our ROM and average ROM stated by Schikowski [23]. A significant difference in the ROM of the atlantoaxial and atlantooccipital joint could not be detected between the breeds included in this study. The only statistically significant difference detectable was smaller axial rotation of the atlantooccipital joint in Chihuahuas in trot. Partially, this finding can be explained by the upright position of the neck in most Chihuahuas, that might reduce the necessity of axial rotations of the CCJ compensatory to trunk rotations. In general, we could not detect increased atlantoaxial or atlantooccipital mobility during locomotion in our clinically sound, but CCJA-predisposed dog breeds Chihuahua and CKCS. This does not rule out, that larger ROM are possible in these dogs during provoked maximal movements or are detectable in an increased number of dogs investigated.

As revealed by Arnold [16], there is minor intervertebral movement of the cervical spine during locomotion compared to extended cervical movements in the sagittal and lateral plane like dorsal or lateral bending [16]. Similarly, the occurring ROM during locomotion measured in our study were well below the maximally possible ROM measured in cadaveric studies and anaesthetised patients [26, 37, 47]. In general, the ROM in almost all rotational DOF was smaller in walk than in trot, although this was only statistically significant for the atlantoaxial joint. This finding supports the hypothesis of Schilling and Carrier [48], that there is effective dynamic muscular stabilisation of the spine in trot in order to provide a swinging spine and effectively transmit propulsive forces from the limbs to the trunk [48, 49]. Any comparison of breed-specific *in vivo* ROM during standardised and controlled head–neck movements requires further studies.

**Fig 11:**
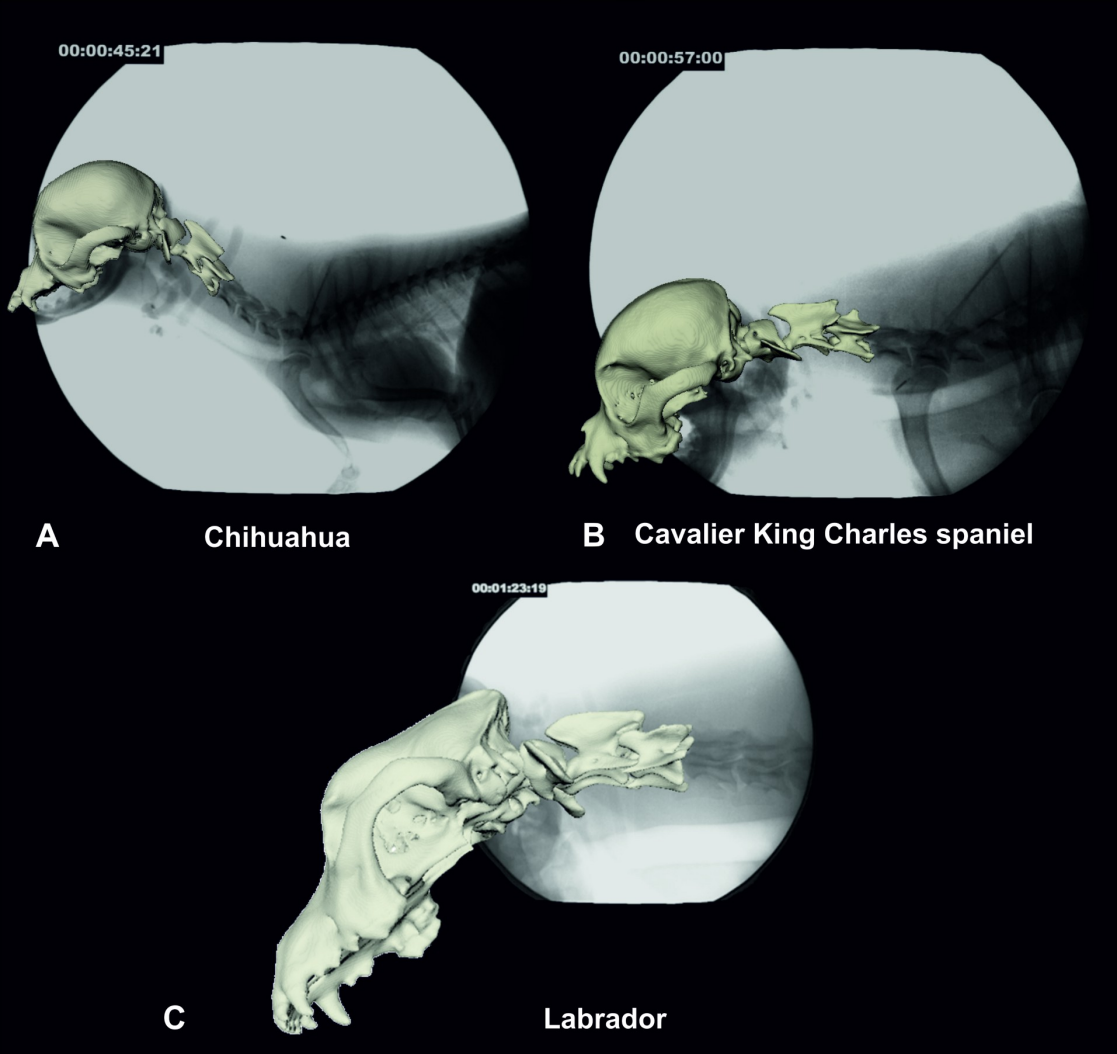
Comparative fluoroscopy images with superimposed bone model of a Chihuahua (A), a CKCS (B) and a Labrador retriever (C). The CKCS and Labrador retriever show a straight neck position in alignment with the withers while the Chihuahua shows an upright neck position and accordingly a steeper atlas angle. Images A and B: 90° camera position, image C: 63° oblique camera position.

A more comparable value than the absolute, unrestricted ROM during locomotion is the mean sagittal rotation of the atlantoaxial joint. Chihuahuas showed the steepest angle of sagittal rotation in the atlantoaxial joint, twice as high as the CKCSs in walk and trot and as the Labrador retrievers in trot, although statistical significance of this observation was not confirmed (S5 Table). Thereby, the large standard deviation of 7.4° in walk and 4.8° in trot in the Chihuahuas (Table 3) has to be noticed. As we found lower mean sagittal atlas angles in the CKCSs, this positional difference does not necessarily indicate a morphology-specific finding in sound small breed dogs with a general predisposition to CCJA. The steep angulation of the atlas in the Chihuahuas is most likely associated with a more upright position of the neck with the cranial base and the base of the vertebral canal having a pointed angle (Fig 11). In Labrador retrievers and most CKCSs, the neck is carried in a rather straight alignment with the thoracolumbar spine and the cranial base and base of the vertebral canal are rather on the same level, causing a flattened atlas angle in relation to the occiput (Fig 11). The steep angulation of the atlas, as observed in most of our Chihuahuas, makes an atlantooccipital overlap in awake and moving dogs with a raised neck very unlikely due to the greater distance between the foramen magnum and dorsal arch of the atlas. The rather flattened posture of the atlantooccipital joint may allow for an intrusion of the dorsal atlas arch into the foramen magnum, also in a standing and moving dog. The same holds true for atlantoaxial dorsal compressive bands. The relevance of both findings must be assessed with caution.

The conformation and proposed limits of flexibility that characterise a sound CCJ [25] were described for dogs under general anaesthesia in a rather extended neck position and, according to our results, mostly reflect the situation in the awake dog. The study of Waschk et al. [25] could not reveal significant differences of their measurements in dogs with extended and mildly flexed head positions, which does not have to be true for awake and moving dogs with a larger variability of the CCJ position. The clivus canal angle, measured exemplarily in dogs of our study population, was obviously smaller (127–154°) than in anaesthetised dogs in dorsal recumbency during CT and MRI examination (168.3 ± 12.5° in dogs without atlantoaxial instability [25]). We accordingly support the recommendations of other authors [15, 40, 50] to use an additional, mildly flexed CCJ position in cross-sectional diagnostics to imitate the naturally occurring CCJ position. Studies on the comparison of clivus canal angles in MRI/CT and in fluoroscopy in standing and moving dogs are necessary to further evaluate abnormal alignment of the CCJ.

Scientific Rotoscoping technique allows highly accurate and minimally invasive measurements, although measurements in the cervical spine are susceptible to be affected by arbitrary head movements. Therefore, Scientific Rotoscoping may be more precise in locations less affected by arbitrary motions, for example the thoracic or lumbar spine. A longer habituation phase on the treadmill over several days in a well-known testing environment in order to reduce arbitrary head movements was not possible in our study, due to the availability of the privately owned dogs. A spatial resolution limit for rotational spine movements of >1.5° has been described while rotational movements of less than 1.5° can only be detected using bone markers [30]. The measurement error in this study is consistent with the measurement error in previous studies using the same methodology [23, 51]. Measurement errors in Scientific Rotoscoping are mainly dependent on the individual dog itself, user experience and positioning of the x-ray cameras [20, 29, 30, 51, 52]. Aiming at an improvement of spatial resolution with bone markers was not an option in this study, due to the use of privately owned dogs. The rotoscoping of the bone model is one of the work steps which is most susceptible for interobserver variation. Therefore, an evaluation of its consistency with two observers was considered more adequate than just comparing the resulting motion data. The interobserver correlation in general delivered low variation with larger differences for certain DOF related to the ROM. The consistency of the animations of the TCCS was twice as high as for the atlantoaxial and atlantooccipital joints (S9 Table). The intervertebral rotations of the atlantoaxial and atlantooccipital joints were predominantly smaller than the movements of the TCCS and were therefore more difficult to rotoscope. A reason for the reduced interobserver consistency concerning the axial rotation of the atlantooccipital joint is the more difficult evaluation of the movement in the fluoroscopy video. The skull of the Labrador retrievers is only partially displayed in the fluoroscopy images due to dog’s size and important landmarks like the teeth are not always displayed. The oblique camera positions in the experimental set up for the Labrador retrievers further complicates the observer’s idea of the movement in space [20]. However, this has important implications for future comparisons between dogs with and without CLM/SM. The personal measurement error was smaller than the interobserver error which underlines a certain observer-dependence but constancy within each observer. A period of training of several weeks was undertaken prior to Scientific Rotoscoping.

Although the study population has been increased compared to our previous study [23], it is still relatively small due to the extremely time consuming data acquisition. Nevertheless, the small study population can be a reason for several non-significant ROM results. Maybe a significant increase of study population with more dogs per breed and more strides analyzed would yet reveal some breed specific differences in motions patterns or ROM we were not able to demonstrate.

## Conclusions

Basic motion patterns of the CCJ and cranial cervical spine are consistent in our study population of clinically sound dog breeds predisposed and not predisposed to CCJA during locomotion. Increased atlantoaxial or atlantooccipital mobility during locomotion could not be confirmed in sound CKCSs and Chihuahuas as representatives of CCJA predisposed dogs. Quantitative measurements of the CCJ obtained in tomographic imaging studies represent the morphology in awake standing and moving dogs. Our results serve as a reference for further studies investigating individuals with clinical signs and confirmed CCJA.

## Supporting information

S1 Table Treadmill speeds and phase normalisation of CKCSs, Chihuahuas and Labrador retrievers in walk and trot. (DOCX)

S2 Table Mean ± standard deviation in % of the timing of directional changes (TOO) within a stride cycle for translations and rotations of the total cranial cervical spine (TCCS). (DOCX)

S3 Table Mean ± standard deviation in % of the timing of directional changes (TOO) within a stride cycle for rotations of the C2/C1 IVJ. (DOCX)

S4 Table Mean ± standard deviation in % of the timing of directional changes (TOO) within a stride cycle for rotations of the C1/skull IVJ. (DOCX)

S5 Table Test for normal distribution and significance of the mean sagittal rotation of the atlantoaxial joint in walk and trot among the breeds. (DOCX)

S6 Table Test for normal distribution of range of motion differences among the breeds. (DOCX)

S7 Table Significance analysis of breed differences in ranges of motion in walk and trot. (DOCX)

S8 Table Test for normal distribution and significance of range of motion differences in walk and trot. (DOCX)

S9 Table Interobserver variability. (DOCX)

S1 Fig Calculation of ROM and average ROM. (DOCX)

S1 File (ZIP)
