# Supplementary material for: Three-dimensional kinematics of the craniocervical junction of Cavalier King Charles Spaniels compared to Chihuahuas and Labrador retrievers
Source: PLoS One. 2023 Jan 17;18(1):e0278665. doi: 10.1371/journal.pone.0278665 (PMC9844835; doi:10.1371/journal.pone.0278665)
Supplement: S1 Table — (DOCX) [file pone.0278665.s001.docx]

**S1 Table: Treadmill speeds and phase normalisation of CKCSs, Chihuahuas and Labrador retrievers in walk and trot.**

| Proband | Treadmill speed [m/s] | | Phase normalisation in walk/trot | |
| --- | --- | --- | --- | --- |
|  | walk | trot | walk | trot |
| CKCS1 | 0.65 | 1.52 | 70/30 | 50/50 |
| CKCS2 | 0.74 | 1.40 | 60/40 | 50/50 |
| CKCS3 | 0.71 | 1.40 | 60/40 | 40/ 60 |
| CKCS4 | 0.50 | 1.10 | 70/30 | 50/50 |
| CKCS5 | 0.65 | - | 70/30 | - |
| CKCS6 | 0.56 | - | 70/30 | - |
| CKCS7 | 0.71 | - | 70/30 | - |
| CKCS8 | 0.68 | - | 70/30 | - |
| CKCS9 | - | 1.10 | - | 50/50 |
| CKCS10 | - | 1.47 | - | 50/50 |
| CKCS11 | - | 1.47 | - | 50/50 |
| CKCS12 | - | 1.47 | - | 50/50 |
| **Total CKCS** | **0.63 ± 0.08** | **1.37 ± 0.16** |  |  |
| Ch1 | 0.39 | 0.73 | 70/30 | 60/40 |
| Ch2 | 0.52 | 0.95 | 60/40 | 50/50 |
| Ch3 | 0.38 | 0.72 | 60/40 | 50/50 |
| Ch4 | 0.32 | 0.72 | 60/40 | 50/50 |
| Ch5 | 0.45 |  | 70/30 |  |
| Ch6 | 0.38 |  | 70/30 |  |
| Ch7 | 0.50 |  | 60/40 |  |
| Ch8 | 0.60 |  | 60/40 |  |
| **Total Chihuahua** | **0.44 ± 0.09** | **0.77 ± 0.12** |  |  |
| L1 | 0.77 | 1.85 | 60/40 | 50/50 |
| L2 | 0.98 | - | 60/40 | - |
| L3 | 1.20 | 1.85 | 60/40 | 50/50 |
| L4 | - | 1.63 | - | 50/50 |
| **Total Labrador** | **0.98 ± 0.18** | **1.78 ± 0.1** |  |  |
