# Supplementary material for: Three-dimensional kinematics of the craniocervical junction of Cavalier King Charles Spaniels compared to Chihuahuas and Labrador retrievers
Source: PLoS One. 2023 Jan 17;18(1):e0278665. doi: 10.1371/journal.pone.0278665 (PMC9844835; doi:10.1371/journal.pone.0278665)
Supplement: S2 Table — (DOCX) [file pone.0278665.s002.docx]

**S2 Table: Mean ± standard deviation in % of the timing of directional changes (TOO) within a stride cycle for translations and rotations of the total upper cervical spine (TUCS).**

|  | Horizontal translations TCCS | | | | | | | |
| --- | --- | --- | --- | --- | --- | --- | --- | --- |
|  | Walk | | | | Trot | | | |
|  | TP1 | TP2 | TP3 | TP4 | TP1 | TP2 | TP3 | TP4 |
| CKCS | 6.76  ± 10.7 | 27.24  ± 10.0 | 57.48  ± 9.0 | 79.21  ± 9.7 | −1.03  ± 4.6 | 22.47  ± 4.3 | 46.15  ± 4.8 | 72.44  ± 4.2 |
| Labrador | 25.90  ± 0.1 | 39.67  ± 7.2 | 68.39  ± 2.8 | 92.33  ± 2.8 | 9.11  ± 2.5 | 33.44  ± 3.4 | 59.56  ± 3.7 | 82.89  ± 3.2 |
| Chihuahua | 16.3  ± 10.5 | 36.0  ± 6.6 | 60.7  ± 7.3 | 87.9  ± 6.1 | 87.22  ± 5.3 | 27.28  ± 16.8 | 38.78  ± 2.7 | 64.33  ± 1.5 |
|  | Vertical translations TCCS | | | | | | | |
|  | Walk | | | | Trot | | | |
|  | TP1 | TP2 | TP3 | TP4 | TP1 | TP2 | TP3 | TP4 |
| CKCS | 8.18  ± 7.4 | 27.65  ± 8.0 | 56.60  ± 10.2 | 78.65  ± 8.0 | 9.57  ± 4.4 | 34.49  ± 3.8 | 58.50  ± 5.6 | 84.78  ± 4.0 |
| Labrador | 8.52  ± 7.3 | 31.98  ± 4.6 | 57.08  ± 4.6 | 80.17  ± 6.8 | 20.28  ± 2.3 | 46.00  ± 0.8 | 69.39  ± 2.2 | 97.57  ± 1.2 |
| Chihuahua | 17.60  ± 7.3 | 39.31  ± 10.6 | 67.64  ± 6.5 | 89.21  ± 6.6 | 7.06  ± 3.6 | 32.42  ± 3.3 | 58.21  ± 4.7 | 82.52  ± 3.6 |
|  | Lateral Translations TCCS | | | | | | | |
|  | Walk | | | | Trot | | | |
|  | TP1 | TP2 | TP3 | TP4 | TP1 | TP2 | TP3 | TP4 |
| CKCS | 40.09  ± 4.1 | 91.15  ± 5.6 | - | - | 30.77  ± 5.6 | 79.64  ± 6.6 | - | - |
| Labrador | 33.63  ± 2.6 | - | - | - | 37.37  ± 2.8 | 79.53  ± 9.3 | - | - |
| Chihuahua | 35.83  ± 7.7 | - | - | - | 27.39  ± 2.9 | 71.88  ± 3.2 | - | - |
|  | Axial rotations TCCS | | | | | | | |
|  | Walk | | | | Trot | | | |
|  | TP1.1 | TP1.2 | TP2.1 | TP2.2 | TP 1 | TP2 | TP3 | TP4 |
| CKCS | 15.25  ± 9.9 | 32.47  ± 7.4 | 66.19  ± 10.7 | 80.04  ± 8.2 | 30.78  ± 7.4 | 79.30  ± 4.6 | - | - |
| Labrador | 33.0  ± 1.0 |  | 86.7  ± 5.8 |  | 40.0 | 86.5 | - | - |
| Chihuahua | - | - | - | - | - | - | - | - |
|  | Lateral rotations TCCS | | | | | | | |
|  | Walk | | | | Trot | | | |
|  | TP1 | TP2 | TP3 | TP4 | TP1 | TP2 | TP3 | TP4 |
| CKCS | 21.0  ± 5.9 | 69.7  ± 15.2 | - | - | 24.94  ± 12.2 | 74.39  ± 13.2 | - | - |
| Labrador | 39.79  ± 3.5 | 80.8  ± 0.2 | - | - | - | - | - | - |
| Chihuahua | 53.1  ± 9.4 | 78.0  ± 9.8 | - | - | - | - | - | - |
|  | Sagittal rotations TCCS | | | | | | | |
|  | Walk | | | | Trot | | | |
|  | TP1 | TP2 | TP3 | TP4 | TP1 | TP2 | TP3 | TP4 |
| CKCS | 17.60  ± 4.6 | 40.75 ±7.6 | 69.07  ± 7.3 | 90.14  ± 3.8 | 24.99  ± 5.0 | 49.81  ± 4.5 | 76.00  ± 4.5 | 98.63  ± 6.5 |
| Labrador | 19.44  ± 1.7 | 46.00  ± 1.8 | 70.50  ± 1.3 | 94.36  ± 1.7 | 43.92  ± 6.6 | 63.42  ± 1.6 | 91.17  ± 4.8 | 9.75  ± 0.3 |
| Chihuahua | 22.9  ± 6.2 | 41.5  ± 4.0 | 73.6  ± 10.7 | 90.5  ± 3.6 | 27.83 ±2.5 | 50.39  ± 3.2 | 76.92  ± 1.4 | 3.6  ± 0.2 |
| breed | Axial rotations TCCS | | | | | | | |
|  | Walk | | | | Trot | | | |
|  | TP1.1 | TP1.2 | TP2.1 | TP2.2 | TP 1 | TP2 | TP3 | TP4 |
| CKCS | 15.25  ± 9.9 | 32.47  ± 7.4 | 66.19  ± 10.7 | 80.04  ± 8.2 | 30.78  ± 7.4 | 79.30  ± 4.6 | - | - |
| Labrador | 33.0  ± 1.0 | - | 86.70  ± 5.8 | - | 40.0 | 86.5 | - | - |
| Chihuahua | - | - | - | - | - | - | - | - |
|  | Lateral rotations TCCS | | | | | | | |
|  | Walk | | | | Trot | | | |
|  | TP1 | TP2 | TP3 | TP4 | TP1 | TP2 | TP3 | TP4 |
| CKCS | 21.0  ± 5.9 | 69.7  ± 15.2 | - | - | 24.94  ± 12.2 | 74.39  ± 13.2 | - | - |
| Labrador | 39.79  ± 3.5 | 80.8  ± 0.2 | - | - | - | - | - | - |
| Chihuahua | 53.1  ± 9.4 | 78.0  ± 9.8 | - | - | - | - | - | - |
|  | Sagittal rotations TCCS | | | | | | | |
|  | Walk | | | | Trot | | | |
|  | TP1 | TP2 | TP3 | TP4 | TP1 | TP2 | TP3 | TP4 |
| CKCS | 17.60  ± 4.6 | 40.75 ±7.6 | 69.07  ± 7.3 | 90.14  ± 3.8 | 24.99  ± 5.0 | 49.81  ± 4.5 | 76.00  ± 4.5 | 98.63  ± 6.5 |
| Labrador | 19.44  ± 1.7 | 46.00  ± 1.8 | 70.50  ± 1.3 | 94.36  ± 1.7 | 43.92  ± 6.6 | 63.42  ± 1.6 | 91.17  ± 4.8 | 109.75  ± 0.3 |
| Chihuahua | 22.9  ± 6.2 | 41.50  ± 4.0 | 73.60  ± 10.7 | 90.50  ± 3.6 | 27.83 ±2.5 | 50.39  ± 3.2 | 76.92  ± 1.4 | 103.6  ± 0.2 |

Averaged for all CKCSs, Labrador retrievers and Chihuahuas in walk and trot. TOO 0% = touchdown of the left hind limb. TOO 100% = subsequent touchdown of the left hindlimb. TP1 = first turning point within the stride cycle, TP2 = second turning point within the stride cycle […], TP1.1 = starting point of a curve deflection when no exact TOO is measurable, TP 1.2 = endpoint of a curve deflection when no exact TOO is measurable. When two turning points are declared, the motion has a monophasic pattern. When four turning points are declared, the motion has a biphasic pattern. Blank field: no uniform TOO measurable.
