# Supplementary material for: Three-dimensional kinematics of the craniocervical junction of Cavalier King Charles Spaniels compared to Chihuahuas and Labrador retrievers
Source: PLoS One. 2023 Jan 17;18(1):e0278665. doi: 10.1371/journal.pone.0278665 (PMC9844835; doi:10.1371/journal.pone.0278665)
Supplement: S3 Table — (DOCX) [file pone.0278665.s003.docx]

**S3 Table: Mean ± standard deviation in % of the timing of directional changes (TOO) within a stride cycle for rotations of the C2/C1 IVJ.**

| Breed | Axial rotations IVJ C2/C1 (atlantoaxial) | | | | | | | |
| --- | --- | --- | --- | --- | --- | --- | --- | --- |
|  | walk | | | | trot | | | |
|  | TP1.1 | TP1.2 | TP2.1 | TP2.2 | TP 1 | TP2 | TP3 | TP4 |
| CKCS | 18.00  ± 7.3 | 27.40  ± 6.6 | 78.10  ± 12.1 | 83.70  ± 12.2 | 9.21  ± 1.5 | 16.59  ± 0.9 | 46.75  ± 1.8 | 59.49  ± 4.2 |
| Labrador | 49.75  ± 4.6 | - | 87.75  ± 5.6 | - | 36.17  ± 2.9 | - | 62.0  ± 4.4 | - |
| Chihuahua | - | - | - | - | - | - | - | - |

| Breed | Sagittal rotations IVJ C2/C1 (atlantoaxial) | | | | | | | | | | | | | | | |
| --- | --- | --- | --- | --- | --- | --- | --- | --- | --- | --- | --- | --- | --- | --- | --- | --- |
|  | walk | | | | | | | | trot | | | | | | | |
|  | TP1.1 | TP1.2 | TP2.1 | TP2.2 | TP3.1 | TP3.2 | TP4.1 | TP4.2 | TP1.1 | TP1.2 | TP2.1 | TP2.2 | TP3.1 | TP3.2 | TP4.1 | TP4.2 |
| CKCS | 6.50  ±2.9 | 16.53  ±1.1 | 24.49 ±5.0 | 36.70 ±42 | 43,78 ±2.8 | 65.78 ±2.7 | 81.18 ±6.5 | 90.47 ±4.6 | 12.67 ±9.2 | 19.67 ±9.8 | 27.75 ±5.3 | 35.13 ±5.4 | 47.60 ±9.1 | 61.60 ±11.0 | 84.00 ±4.0 | 97.50 ±2.5 |
| Labrador | 5.75 ±3.3 | 14.17  ±2.2 | 24.69 ±6.8 | 31.04 ±6.7 | 49.11 ±4.4 | 57.22 ±1.9 | 77.39 ±2.1 | 86.00 ±0.0 | 11.17 ±3.2 | 22.84 ±5.3 | 43.87 ±1.5 | 51.00 ±2.2 | 54.50 ±3.7 | 61.00 ± 5.1 | 77.00 ±6.7 | 85.50  ±3.7 |
| Chihuahua | - |  | - |  | - |  | - |  | - |  | - |  | - |  | - |  |

Averaged for all CKCSs, Labrador retrievers and Chihuahuas in walk and trot. TOO 0% = touchdown of the left hind limb. TOO 100%= subsequent touchdown of the left hindlimb. TP1 = first turning point within the stride cycle, TP2 = second turning point within the stride cycle […], TP1.1. = starting point of a curve deflection when no exact TOO is measurable, TP 1.2 = endpoint of a curve deflection when no exact TOO is measurable. When two turning points are declared, the motion has a monophasic pattern. When four turning points are declared, the motion has a biphasic pattern. Blank field: no uniform TOO measurable.
