# Supplementary material for: Three-dimensional kinematics of the craniocervical junction of Cavalier King Charles Spaniels compared to Chihuahuas and Labrador retrievers
Source: PLoS One. 2023 Jan 17;18(1):e0278665. doi: 10.1371/journal.pone.0278665 (PMC9844835; doi:10.1371/journal.pone.0278665)
Supplement: S4 Table — (DOCX) [file pone.0278665.s004.docx]

**S4 Table: Mean ± standard deviation in % of the timing of directional changes (TOO) within a stride cycle for rotations of the C1/skull IVJ.**

| Sagittal rotation IVJ C2C1/skull (atlantooccipital) | | | | | | | | | | | | | | | | |
| --- | --- | --- | --- | --- | --- | --- | --- | --- | --- | --- | --- | --- | --- | --- | --- | --- |
| Breed | Walk | | | | | | | | Trot | | | | | | | |
|  | TP1.1 | TP1.2 | TP2.1 | TP2.2 | TP3.1 | TP3.2 | TP4.1 | TP4.2 | TP1.1 | TP1.2 | TP2.1 | TP2.2 | TP3.1 | TP3.2 | TP4.1 | TP4.2 |
| CKCS | 9.56 ±1.8 | 16.78 ±2.7 | 22.82 ±4.7 | 35.60 ±3.6 | 50.34 ±10.2 | 60.19 ±9.6 | 73.26 ±7.7 | 83.24 ±6.4 | 7.83 ±2.0 | 24.33 ±1.7 | 29.70 ±10.2 | 44.57 ±6.6 | 44.20 ±0.8 | 63.70 ±11.3 | 79.70 ±6.6 | 92.70 ±7.5 |
| Labrador | 3.17 ±1.2 | 15.96 ±2.7 | 22.28 ±4.5 | 38.15 ±0.35 | 50.67 ± 2.87 | 68.25 ±0.9 | 75.58 ±3.8 | 85.08 ±1.5 | 11.60 ± 0.6 | 26.00 ±8.0 | 29.00 ±11.2 | 45.67 ±10.1 | 53.25 ±10.8 | 67.25 ± 6.8 | 82.17 ±7.6 | 64.61 ±40.0 |
| Chihuahua | 11.52 ±4.6 |  | 40.90 ±3.6 |  | 64.85 ±5.3 |  | 89.08 ±2.7 |  | - |  | - |  | - |  | - |  |

Averaged for all CKCSs, Labrador retrievers and Chihuahuas in walk and trot. TOO 0% = touchdown of the left hind limb. TOO 100%= subsequent touchdown of the left hindlimb. TP1 = first turning point within the stride cycle, TP2 = second turning point within the stride cycle […], TP1.1. = starting point of a curve deflection when no exact TOO is measurable, TP 1.2 = endpoint of a curve deflection when no exact TOO is measurable. When two turning points are declared, the motion has a monophasic pattern. When four turning points are declared, the motion has a biphasic pattern. Blank field: no uniform TOO measurable.
