# Supplementary material for: Three-dimensional kinematics of the craniocervical junction of Cavalier King Charles Spaniels compared to Chihuahuas and Labrador retrievers
Source: PLoS One. 2023 Jan 17;18(1):e0278665. doi: 10.1371/journal.pone.0278665 (PMC9844835; doi:10.1371/journal.pone.0278665)
Supplement: S5 Table — (DOCX) [file pone.0278665.s005.docx]

**S5 Table: Test for normal distribution and significance of the average sagittal rotation of the atlantoaxial joint in walk and trot among the breeds.**

|  | Shapiro–Wilk test  p-value | Kruskal-Wallis Test  p-value | One-way ANOVA  p-value |
| --- | --- | --- | --- |
| Walk | 0.047* | 0.182 | - |
| Trot | 0.3 | - | 0.189 |

* p<0.05
