# Supplementary material for: Three-dimensional kinematics of the craniocervical junction of Cavalier King Charles Spaniels compared to Chihuahuas and Labrador retrievers
Source: PLoS One. 2023 Jan 17;18(1):e0278665. doi: 10.1371/journal.pone.0278665 (PMC9844835; doi:10.1371/journal.pone.0278665)
Supplement: S6 Table — (DOCX) [file pone.0278665.s006.docx]

**S6 Table: Test for normal distribution of range of motion differences among the breeds.**

| Joint | DOF | p-value | p-value |
| --- | --- | --- | --- |
|  |  | walk | trot |
| Atlantoaxial | Sagittal rotation | 0.072 | 0.835 |
| Atlantoaxial | Axial rotation | 0.223 | 0.083 |
| Atlantoaxial | Lateral rotation | 0.199 | 0.244 |
| Atlantooccipital | Axial rotation | 0.355 | 0.023* |
| Atlantooccipital | Lateral rotation | 0.353 | 0.548 |
| Atlantooccipital | Sagittal rotation | 0.035 | 0.035 |

P-values in walk and trot for all rotational degrees of freedom of the atlantoaxial and atlantooccipital joints. *: p<0.05
