# Supplementary material for: Three-dimensional kinematics of the craniocervical junction of Cavalier King Charles Spaniels compared to Chihuahuas and Labrador retrievers
Source: PLoS One. 2023 Jan 17;18(1):e0278665. doi: 10.1371/journal.pone.0278665 (PMC9844835; doi:10.1371/journal.pone.0278665)
Supplement: S7 Table — (DOCX) [file pone.0278665.s007.docx]

**S7 Table: Significance analysis of breed differences in ranges of motion in walk and trot** **for all rotational degrees of freedom of the atlantoaxial and atlantooccipital joints.**

| Joint | DOF | p-value | p-value | post-hoc test for significant results |
| --- | --- | --- | --- | --- |
|  |  | Walk | Trot |  |
| Atlantoaxial | Sagittal rotation | 0.072 | 0.063 |  |
| Atlantoaxial | Axial rotation | 0.191 | 0.282 |  |
| Atlantoaxial | Lateral rotation | 0.143 | 0.194 |  |
| Atlantooccipital | Axial rotation | 0.096 | 0.026* | Labrador – CKCS: 0.656  Labrador - Chihuahua: 0.042*  CKCS – Chihuahua: 0.047* |
| Atlantooccipital | Lateral rotation | 0.168 | 0.842 |  |
| Atlantooccipital | Sagittal rotation | 0.325 | 0.429 |  |

* p<0.05
