# Supplementary material for: Three-dimensional kinematics of the craniocervical junction of Cavalier King Charles Spaniels compared to Chihuahuas and Labrador retrievers
Source: PLoS One. 2023 Jan 17;18(1):e0278665. doi: 10.1371/journal.pone.0278665 (PMC9844835; doi:10.1371/journal.pone.0278665)
Supplement: S8 Table — (DOCX) [file pone.0278665.s008.docx]

**S8 Table: Test for normal distribution and significance of range of motion differences in walk and trot for all rotational degrees of freedom of the atlantoaxial and atlantooccipital joint.**

| Joint | DOF | Shapiro–Wilk test  p-value | Mann–Whitney U-test / t-test  p-value |
| --- | --- | --- | --- |
| Atlantoaxial | Sagittal rotation | 0.064 | 0.043* |
| Atlantoaxial | Axial rotation | 0.009* | 0.012* |
| Atlantoaxial | Lateral rotation | 0.046* | 0.009* |
| Atlantooccipital | Axial rotation | 0.011* | 0.488 |
| Atlantooccipital | Lateral rotation | 0.006* | 0.116 |
| Atlantooccipital | Sagittal rotation | 0.004* | 0.169 |

* p<0.05
