# Supplementary material for: Three-dimensional kinematics of the craniocervical junction of Cavalier King Charles Spaniels compared to Chihuahuas and Labrador retrievers
Source: PLoS One. 2023 Jan 17;18(1):e0278665. doi: 10.1371/journal.pone.0278665 (PMC9844835; doi:10.1371/journal.pone.0278665)
Supplement: S9 Table — (DOCX) [file pone.0278665.s009.docx]

**S9 Table: Interobserver variability.**

| **Axis** | **C3/C3 translation** | **C3/C3 rotation** | **C2/C1 rotation** | **C1/skull rotation** |
| --- | --- | --- | --- | --- |
| x abs. (cm/°) | 0.14 ± 0.10 | 1.75 ± 1.22 | 0.95 ± 0.75 | 1.65 ± 1.26 |
| x rel. (%) | 5.98 | 12.93 | 24.58 | 31.70 |
| y abs. (cm/°) | 0.16 ± 0.10 | 1.28 ± 0.84 | 1.72 ± 1.28 | 1.74 ± 1.16 |
| y rel. (%) | 6.77 | 9.04 | 17.41 | 26.57 |
| z abs. (cm/°) | 0.14 ± 0.10 | 0.89 ± 0.61 | 1.33 ± 0.85 | 1.45 ± 1.15 |
| z rel. (%) | 4.27 | 7.68 | 23.01 | 16.15 |
| **total abs. (**cm/°) | **0.15 ± 0.0** | **1.31 ± 0.4** | **1.33 ± 0.31** | **1.61 ± 0.12** |
| **total rel.** (%) | **5.67 ± 1.04** | **9.88 ± 2.3** | **21.67 ± 3.08** | **24.80 ± 6.47** |

Mean and standard deviation of the absolute (abs.) difference in cm for translations and in degrees for rotations and mean relative difference related to the range of motion (rel.) in % between the motion curves of the authors MN and LS for three Labrador retrievers in walk. X, y and z indicate the coordinate axis along which the movement occurs. C3/C3 indicates IVJ C3/C4 and represents the uppermost hierarchical point of the bone model.
