# Supplementary material for: Three-dimensional kinematics of the craniocervical junction of Cavalier King Charles Spaniels compared to Chihuahuas and Labrador retrievers
Source: PLoS One. 2023 Jan 17;18(1):e0278665. doi: 10.1371/journal.pone.0278665 (PMC9844835; doi:10.1371/journal.pone.0278665)
Supplement: S1 Fig — (DOCX) [file pone.0278665.s010.docx]

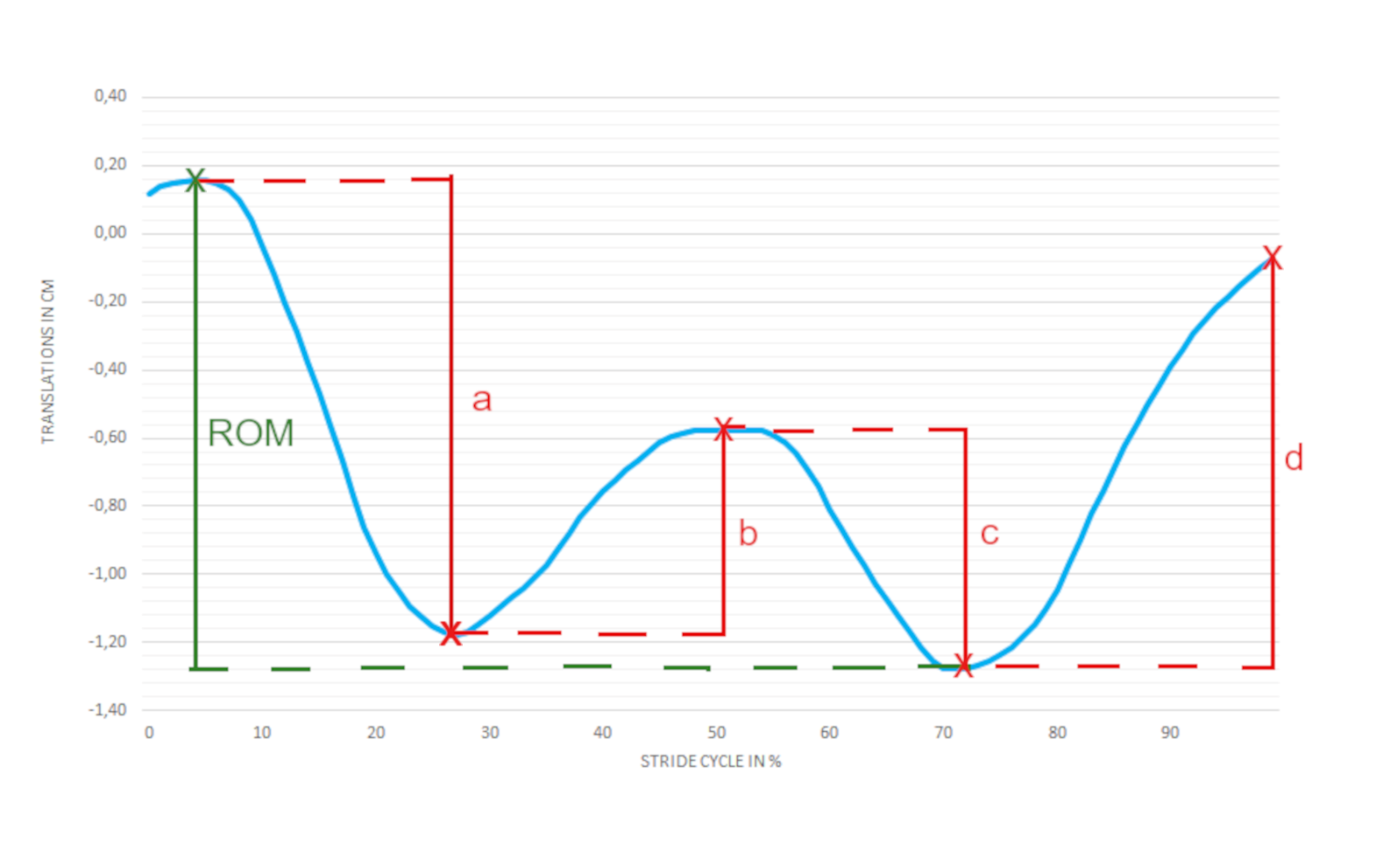


**S1 Figure: Calculation of ROM and average ROM.** ROM was calculated in this study, average ROM was calculated by Schikowski [22]. X-axis represents one complete stride cycle from touchdown (0%) to subsequent touchdown (100%) of the left hind limb. Y-axis represents the motion amplitude in cm The blue line represents an exemplary motion curve during one stride cycle. The solid green line represents the ROM in one DOF of the stride depicted. An arithmetic mean of the curve deflection of all solid red lines a-d represents the average ROM.
